# Zebrafish Cancer Predisposition Models

**DOI:** 10.3389/fcell.2021.660069

**Published:** 2021-04-27

**Authors:** Kim Kobar, Keon Collett, Sergey V. Prykhozhij, Jason N. Berman

**Affiliations:** ^1^Children’s Hospital of Eastern Ontario Research Institute, Ottawa, ON, Canada; ^2^Department of Cellular and Molecular Medicine, University of Ottawa, Ottawa, ON, Canada; ^3^Department of Pediatrics, University of Ottawa, Ottawa, ON, Canada

**Keywords:** zebrafish, cancer predisposition, genetic models, cancer, model organism, p53

## Abstract

Cancer predisposition syndromes are rare, typically monogenic disorders that result from germline mutations that increase the likelihood of developing cancer. Although these disorders are individually rare, resulting cancers collectively represent 5–10% of all malignancies. In addition to a greater incidence of cancer, affected individuals have an earlier tumor onset and are frequently subjected to long-term multi-modal cancer screening protocols for earlier detection and initiation of treatment. *In vivo* models are needed to better understand tumor-driving mechanisms, tailor patient screening approaches and develop targeted therapies to improve patient care and disease prognosis. The zebrafish (*Danio rerio*) has emerged as a robust model for cancer research due to its high fecundity, time- and cost-efficient genetic manipulation and real-time high-resolution imaging. Tumors developing in zebrafish cancer models are histologically and molecularly similar to their human counterparts, confirming the validity of these models. The zebrafish platform supports both large-scale random mutagenesis screens to identify potential candidate/modifier genes and recently optimized genome editing strategies. These techniques have greatly increased our ability to investigate the impact of certain mutations and how these lesions impact tumorigenesis and disease phenotype. These unique characteristics position the zebrafish as a powerful *in vivo* tool to model cancer predisposition syndromes and as such, several have already been created, including those recapitulating Li-Fraumeni syndrome, familial adenomatous polyposis, RASopathies, inherited bone marrow failure syndromes, and several other pathogenic mutations in cancer predisposition genes. In addition, the zebrafish platform supports medium- to high-throughput preclinical drug screening to identify compounds that may represent novel treatment paradigms or even prevent cancer evolution. This review will highlight and synthesize the findings from zebrafish cancer predisposition models created to date. We will discuss emerging trends in how these zebrafish cancer models can improve our understanding of the genetic mechanisms driving cancer predisposition and their potential to discover therapeutic and/or preventative compounds that change the natural history of disease for these vulnerable children, youth and adults.

## Introduction

### Cancer Predisposition Genes

Tumor-specific mutations that cause cancer are usually somatic and are cumulatively acquired over a lifetime, which explains the greater cancer prevalence in the older demographic. In contrast, germline mutations in certain genes, termed cancer predisposition genes (CPGs), are associated with an increased cancer risk. Germline mutations in CPGs often lead to hereditary cancer syndromes, which represent 5–10% of all malignancies and typically have a much earlier tumor onset than their sporadic counterparts (Garber and Offit, 2005). Despite the lack of a generally accepted CPG definition, one study identified 114 CPGs as the genes whose rare mutations increase the relative risk of cancer > 2 fold in at least 5% of individuals (Rahman, 2014). Another extensive study analyzed tumors from 10,389 individuals across 33 different cancer types and identified 152 CPGs using CharGer (Characterization of Germline Variants), an automatic variant classification pipeline they developed that ranks the pathogenicity of a certain mutation (Huang et al., 2018).

Unsurprisingly, many frequently mutated cancer driver genes in sporadic cancers have also been classified as CPGs, such as *TP53, APC, BRCA1/2, ATM*, and *NF1*, and as such, the knowledge gained by examining the role of germline CPG mutations will help shed light on the mechanisms of tumor formation for both sporadic and inherited cancers (Lu et al., 2015; Zhang et al., 2015). Additionally, 115 novel ultra-rare cancer-exclusive variants have been associated with known and candidate CPGs (Rasnic et al., 2020). Somatic mutations in 21 of these candidate CPGs were identified in 35% of cancers from 10,953 patients and were associated with a significantly decreased median survival time. This study shows the relevance of CPGs to the broader cancer research field and demonstrates the need to expand the list of known CPGs to assist with personalized diagnostic approaches and prognosis of cancers as well as to better understand the biological mechanisms of tumorigenesis to inform novel targeted therapeutic strategies.

### Zebrafish as a Cancer Disease Model System

**FIGURE 1:**
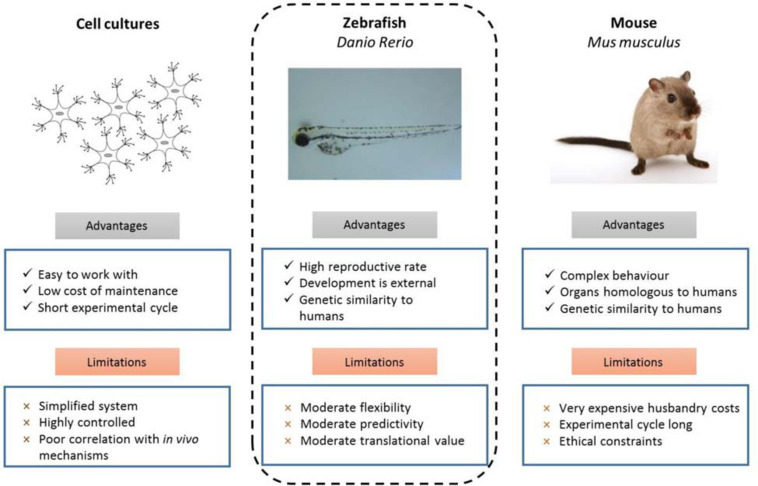
Experimental advantages and limitations of commonly used model organisms (d’Amora and Giordani, 2018).

There are several traditional model systems commonly used to study cancer, including cell culture based, zebrafish and mouse models. These models all have their own unique advantages and limitations (Figure 1) which can be leveraged in different ways to contribute to cancer and developmental biology as well as to human disease research.

The many advantages of the zebrafish position it as a powerful *in vivo* model, especially for preclinical genetic studies to investigate cancer predisposition (Figure 1). Zebrafish share a high degree of genetic conservation with humans, and tumors that develop in zebrafish are histologically similar to their human counterparts. Zebrafish larvae (and some adult strains such as *casper* White et al., 2008) are transparent, which enables live high-resolution imaging to track single cells and visualize tissues, as well as for large-scale and non-invasive tumor screening studies which is much more feasible than in a mouse model. The high fecundity of zebrafish combined with external fertilization and rapid development to sexual maturity makes the creation of new disease models by genome editing rapid and inexpensive in comparison to the much lower number of offspring, longer time to reach sexual maturity, and higher housing costs of rearing mice. The specificity and adaptability of genome editing technologies available to zebrafish researchers has grown tremendously in the recent years and facilitates both the generation of representative models for specific disease-causing mutations and forward genetic screens to identify genes and pathways involved in cancer predisposition.

### Genome Engineering Strategies for Cancer Predisposition Genetic Models in Zebrafish

Genome engineering technologies have been heavily utilized in zebrafish disease modelling. A recent review catalogued and thoroughly discussed available technologies, and we encourage readers to consult this review for information on forward genetic screens using chemical mutagens, TILLING (Targeting Induced Local Lesions IN Genomes), transgenic techniques, gene editing using Zinc Finger Nucleases (ZFN), TAL-effector Endonucleases (TALEN) and Clustered Regularly Interspaced Palindromic Repeats (CRISPR)/Cas9 strategies (Raby et al., 2020).

Recent advances have expanded the range of available CRISPR nucleases, guide RNA design tools and other emerging technologies available to edit the zebrafish genome (Prykhozhij et al., 2018a; Liu et al., 2019). Off-target gene editing is a frequent problem when using CRISPR nucleases and this issue has been recently addressed both by the development of more sophisticated guide RNA design tools (Prykhozhij et al., 2018a; Liu et al., 2019) as well as by generating zebrafish-optimized vectors encoding HiFi Cas9 (Vakulskas et al., 2018) and highly specific but less efficient hypaCas9 (Chen et al., 2017) and testing them as mRNAs as well as commercial wild-type and HiFi Cas9 proteins using the same strategies (Prykhozhij et al., 2020).

Generating point mutants efficiently and precisely is one of the most important areas for optimizing genome editing technologies. The use of CRISPR/Cas9 and chemically synthesized oligonucleotides has been the earliest and primary approach to introduce point mutations into the zebrafish genome. We have optimized this technology in zebrafish to generate cancer-relevant point mutants in the *tp53* gene (Prykhozhij et al., 2018b) and have reviewed other studies which applied similar techniques to introduce specific knock-in mutations (Prykhozhij and Berman, 2018). The limitations of this technology are that the double strand breaks and DNA repair machinery are still required for mutant generation, thus limiting efficiency to a maximum of < 10%. CRISPR/Cas9 point mutation knock-ins with enzymatically-synthesized long single-stranded DNA of 300-500 nucleotides as templates have recently been shown to have higher efficiencies in zebrafish compared to shorter oligonucleotides or double-stranded RNA molecules (Bai et al., 2020), but the efficiencies were measured based on single embryo phenotypes, which is prone to over-estimates. The zLOST (zebrafish long single-stranded DNA template) method is directly analogous to a similar Easi-CRISPR method in mice (Miura et al., 2018).

Among newer technologies for introducing precise mutations are base editors and prime editing, which both remove the need for double-strand breaks (DSB) and template oligonucleotides. Base editors use enzymatically inactive dCas9 fused to deaminase enzymes to mediate mutagenesis in defined parts of guide RNA recognition sites (reviewed in Liu et al., 2019). However, base editors are limited by both the inability of one molecule to introduce different types of mutations and a narrow but not completely defined region of activity. The most anticipated technology to be adopted for use in zebrafish is prime editing as developed by the lab of David Liu (Anzalone et al., 2019), who engineered a fusion of a Cas9 nickase with a reverse transcriptase enzyme to perform edits based on the information encoded in a prime editor guide RNA (pegRNA). The basic mechanism of prime editing consists of the following steps: Cas9 nickase cuts the strand opposite from its pegRNA recognition site, the primer-binding site of pegRNA binds to the released single-strand region of target DNA, and the reverse transcriptase enzyme copies the edited sequence from the pegRNA into the single strand of DNA which is then repaired with the edit being stably introduced into the DNA. In another strategy, denoted PE3b, the nickase with the regular guide RNA cuts the unedited strand thus promoting repair using the edited DNA strand (Anzalone et al., 2019).

Additionally, the generation of true null mutants in zebrafish is important for robust gene function analysis. While analyzing the transcriptional adaptation to indel mutants, El-Brolosy identified a strategy of deleting the proximal promoter and the first exon to generate “RNA-less” mutants failing to transcribe the gene (El-Brolosy et al., 2019). This strategy is likely highly robust, based on the published data and our own observations, but its utility has been questioned for some long non-coding RNAs (lncRNA), whose transcription was not fully blocked or altered by RNA-less deletions (Lavalou et al., 2019). Instead, insertion of a poly-adenylation signal was more effective for the *malat* lncRNA gene. GeneWeld is a CRISPR/Cas9 and microhomology-based gene insertion technology typically used for protein fusion gene generation (Wierson et al., 2020) that can be easily used to insert a fluorescent protein tag or another insert coupled with a stop-codon and a poly-adenylation signal at the 5′ end of a protein-coding gene thus disrupting it coupled with a fluorescent marker. Moreover, GeneWeld allows for a transgenic marker such as fluorescent protein expression in the lens or heart thus marking the mutant allele.

## Studies of CPGs in Zebrafish: Developmental and Cancer Predisposition Models

For this review, we focused on zebrafish models of cancer predisposition based on CPGs from published lists (Rahman, 2014; Huang et al., 2018), many of which are implicated in cancer predisposition syndromes such as Li-Fraumeni syndrome (LFS), familial adenomatous polyposis (FAP), Cowden syndrome, Peutz-Jeghers syndrome (PJS), tuberous sclerosis complex (TSC), RASopathies, and certain hematological disorders. We will also discuss CPG mutations that, although not directly linked to a syndrome, are associated with DNA repair and genome stability and thus increase the risk of developing tumors. We have summarized the observations from current zebrafish cancer predisposition models (Supplementary Table 1) and classified all CPGs according to their primary functional category, highlighting those where significant zebrafish studies exist (Table 1).

**TABLE 1:** Broad and non-exclusive functional categories of CPG proteins.

Functional category	Proteins
DNA Repair/Genome stability	**ATM**, **ATR**, **ATRX**, BAP1, BLM, BRCA1, **BRCA2**, **BRIP1**, BUB1B, DDB2, **CHEK2**, **DICER1**, DROSHA, DIS3L2, ERCC1, ERCC2, ERCC3, **ERCC4**, **FANCD2**, **FANCE**, **FANCF**, **FANCA**, **FANCC**, **FANCG**, **FANCM**, **FANCI**, **FANCL**, POLD1, POLE, POLH, **MLH1**, **MSH2**, **MSH6**, MUTYH, NBN, NTHL1, **PALB2**, PMS1, PMS2, **RAD51C**, RAD51D, **RAD51**, RECQL, RECQL4, **SPRTN**, **TP53**, **VHL**, WRN, XPA, XPC
Cell signaling	ALK, **APC**, AXIN2, BMPR1A, BRAF, DOCK8, EGFR, FLCN, GPC3, **HRAS**, ITK, KIT, **KRAS**, MAP2K1, MAP2K2, MPL, **NF1**, NF2, NRAS, **PDGFRA**, PRKAR1A, PTCH1, **PTEN**, **PTPN11**, RAF1, RET, RHBDF2, SH2B3, SH2D1A, SHOC2, SMAD4, SOS1, STAT3, **STK11**, SUFU, TGFBR1, TMEM127, TNFRSF6, **TSC1**, **TSC2**, TSHR
Transcriptional regulation and epigenetic modification	CDC73, **CEBPA**, CTR9, ETV6, **GATA2**, HNF1A, JMJD1C, LMO1, MAX, MEN1, MET, MITF, PAX5, PHOX2B, PRDM9, **RUNX1**, SETBP1, SMARCA4, SMARCB1, SMARCE1, SRY, WT1
Metabolism	FH, FAH, GBA, HMBS, MTAP, SDHA, SDHAF2, SDHC, SDHD, UROD
Cell adhesion and extracellular matrix	CDH1, COL7A1, EXT1, EXT2, EPCAM, GJB2
Ubiquitination and protein degradation	**CBL**, CYLD, PRSS1, SERPINA1, TRIM37
Telomere maintenance	**DKC1**, **NOP10**, NHP2, POT1, **TERT**
Cell cycle machinery and regulation	CDK4, CDKN1B, CDKN2A, CDKN1C, **RB1**
Ribosome biogenesis	**DKC1**, NHP2, **NOP10**, **SBDS**
Membrane transport	ABCB11, HFE, SLC25A13
Immunity	PRF1, ELANE
Mitochondrial RNA processing	RMRP
Cytoskeleton regulation	**WASp**

*Proteins marked in Bold have been studied in zebrafish in the context of cancer predisposition or developmental phenotypes.*

### Li-Fraumeni Syndrome

**FIGURE 2:**
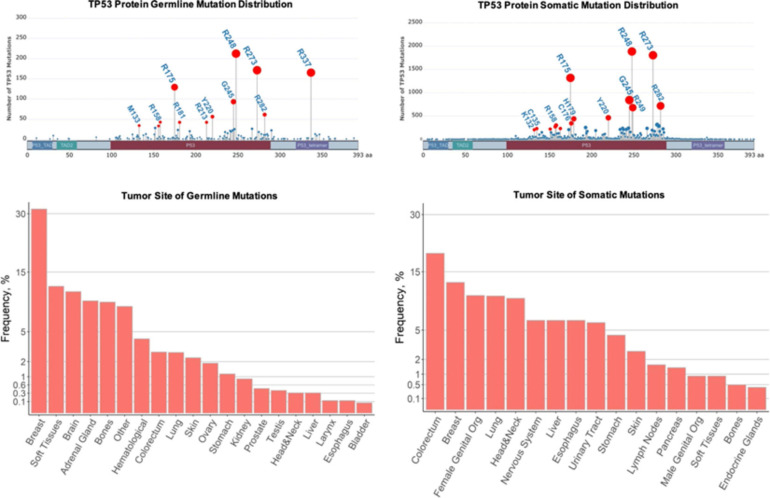
Comparison of germline and sporadic *TP53* mutations found in human cancers. Top row: Germline and somatic missense mutations have been mapped to specific *TP53* residues and domains. The frequencies of the mutations in selected hot-spot residues are indicated by the height of lollipop graph elements and the size of their circles. The circles of selected mutations are highlighted in red and the residues are labeled. The total numbers of germline and somatic mutations recorded are different in IARC TP53 datasets. Bottom row: Tissues associated with tumors due to germline and somatic *TP53* mutations. The frequencies are represented as percentages of the total recorded tumors. In the plot, these percentages are scaled by the square root to visualize less frequent tissues. Plots were constructed using G3viz and ggplot2 R packages based on the IARC TP53 database (https://p53.iarc.fr/) after limiting the datasets to the missense mutations.

Li-Fraumeni syndrome is a hereditary cancer predisposition disorder that albeit rare, is one of the most recognized due to the high penetrance and range of associated tumors. Affected individuals develop a spectrum of malignancies (most commonly breast cancers, bone and soft tissue sarcomas, adrenocortical carcinomas, brain tumors, and leukemia) that typically arise much earlier than their sporadic counterparts (Li et al., 1988; Malkin, 2011; Figure 2). More than 30% of LFS patients develop cancer during childhood and adolescence, and those that survive face an 83-fold relative risk of developing additional malignancies (Hisada et al., 1998). Germline mutations in *TP53* have been identified as the causative event in 70–80% of individuals with LFS (Malkin, 1993). *TP53* is the most commonly mutated gene in all cancers and its corresponding protein, p53, is a potent tumor suppressor that orchestrates the response to cellular stress, such as DNA damage, by activating numerous target genes that induce cell cycle arrest, inhibit proliferation, and promote apoptosis among several other cellular processes (Cadwell and Zambetti, 2001). The most frequent *TP53* mutations found in both LFS and sporadic tumors are missense mutations clustered in several hotspot regions in the DNA binding domain at codons 175, 245, 248, 273, and 282, although germline and somatic mutations present at different frequencies (Figure 2). However, the wide spectrum of tumors that develop and the varying time to onset cannot be simply attributed to *TP53* mutations but may be partially explained by a number of cooperating factors including copy number variants, shortened telomeres, and somatic mutations in modifier genes (Bond et al., 2004; Tabori et al., 2007; Shlien et al., 2008).

*TP53* is highly conserved in zebrafish, especially in the protein DNA-binding domain where ∼90% of the mutations identified in human tumors are found (Hainaut and Pfeifer, 2016). Additionally, zebrafish and mammals share induction of the same p53 target genes (e.g., *cdkn1a*, *cycg1*, *gadd45a/b*, *mdm2*, *bax1*, *puma*, and *noxa*) following ionizing radiation (IR), which is used to induce DNA damage and trigger p53 pathway activation (Parant et al., 2010). Unsurprisingly, several zebrafish *tp53* mutants have been developed to investigate both sporadic cancers and LFS. One of the initial approaches used to create *tp53* point mutants was through *N*-ethyl-*N*-nitrosourea (ENU) targeted mutagenesis. This strategy was employed to generate two zebrafish *tp53* mutants with missense mutations in the DNA-binding domain, *tp53*^*N168K*^ and *tp53*^*M214K*^, equivalent to human *TP53*^*N200K*^ and *TP53*^*M246K*^, the latter being proximal to several hotspot mutations (Berghmans et al., 2005). These mutants were resistant to IR-induced apoptosis, indicating disrupted p53 signaling; however, the *tp53*^*N168K*^ mutants were only capable of this phenomenon when raised at 37°C instead of the normal zebrafish homeostatic temperature of 28.5°C. The *tp53*^*M214K*^ mutants developed malignant peripheral nerve sheath tumors (MPNSTs) with approximately 28% tumor incidence by 16.5 months. However, a low tumor penetrance combined with a lack of tumor development in heterozygous mutants indicate that this mutant does not completely recapitulate the LFS phenotype (Berghmans et al., 2005).

Another zebrafish mutant, *tp53*^*I166T*^, was identified by screening the progeny of ENU-mutagenized zebrafish for the IR-induced apoptosis resistance phenotype (Parant et al., 2010). The location of this mutation is analogous to human codon 195, which is also implicated in several sporadic cancers (Kanda et al., 2013; Mullany et al., 2015). *tp53*^*I166T*^ mutants have characteristics of the LFS phenotype including highly penetrant tumorigenesis, especially sarcomas, and dominant negative activity of mutant p53 (Parant et al., 2010). Both the *tp53*^*I166T*^ homozygous and heterozygous mutants developed tumors and the *tp53*^+/I166T^ mutant tumors displayed a high rate of loss of heterozygosity (LOH) of the wild-type *tp53* allele, a characteristic frequently observed in LFS tumors. Mutants experienced aggressive tumorigenesis, with the first tumor appearing by 9 months and overall a dismal 50% survival rate by 15 months. However, the *tp53*^*I166T*^ mutation was characterized as a loss of function (LOF) mutation in contrast to gain-of-function (GOF) mutations that are more commonly found in both LFS and sporadic tumors (Parant et al., 2010).

To further investigate the effect of p53 loss on tumorigenesis, a *tp53* knockout zebrafish mutant was engineered using TALEN endonucleases (Ignatius et al., 2018). Although most LFS mutations are missense mutations, a small subset of LFS patients possess deletions at the *TP53* locus. The *tp53*^–/–^ fish are resistant to IR-induced apoptosis and developed a wider spectrum of malignancies than the *tp53*^*M214K*^ and *tp53*^*I166T*^ mutants, including MPNSTs, angiosarcomas, germ cell tumors, and a natural killer cell-like leukemia. Homozygous *tp53*^–/–^ mutants developed tumors as early as 5 months with a 37% tumor incidence by 12 months. Heterozygous *tp53*^+/–^ mutants also developed tumors, although infrequently, suggesting LOH also contributes to tumorigenesis in these mutants.

Recently, our group optimized the oligonucleotide-based CRISPR/Cas9 point mutation insertion strategy to generate specific knock-in mutations and employed this strategy to engineer a zebrafish *tp53*^*R217H*^ mutant, equivalent to the human GOF *TP53*^*R248H*^ mutation, representing one of the most frequent hotspot mutations found in both LFS and sporadic tumors (Prykhozhij et al., 2018b). Further characterization of these mutants will help shed light on the specific mechanisms and pathways involved in LFS.

### CPGs in the Wnt/β-Catenin and PI3K/Akt/mTOR Pathways

**FIGURE 3:**
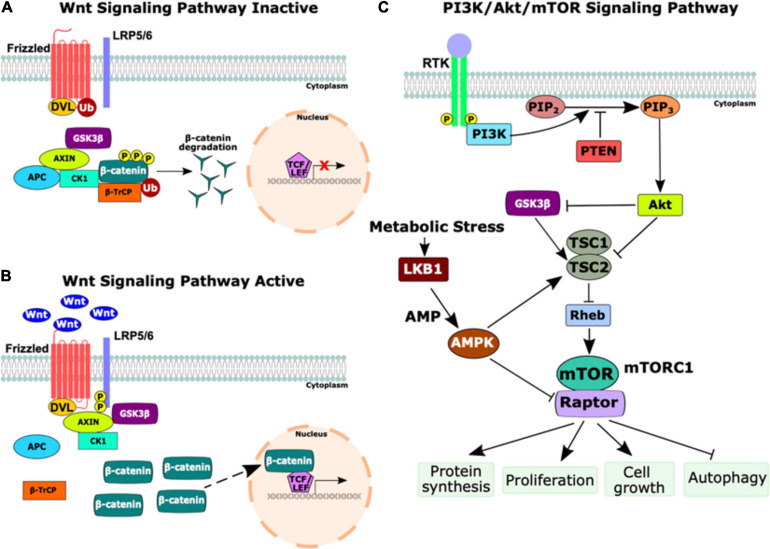
Schematic of the Wnt and PI3K/Akt/mTOR signaling pathways. **(A)** The inactive state of the Wnt signaling pathway. In the absence of Wnt ligands, the destruction complex (Axin, adenomatous polyposis coli (APC), casein kinase 1 (CK1), and GSK3β) phosphorylates β-catenin, marking it for ubiquitination (Ub) by beta-transducin repeat-containing protein (β-TrCP). β-catenin is degraded by proteasomes, thus preventing transcription of Wnt target genes. **(B)** The activation of the Wnt signaling pathway occurs upon the binding of Wnt ligands to Frizzled receptors. Next, CK1 and GSK3β phosphorylate the low-density lipoprotein receptor-related protein 5/6 (LRP5/6) co-receptor, activating DVL, which inhibits the destruction complex and prevents the degradation of β-catenin. Stabilized β-catenin accumulates in the cytoplasm and translocates to the nucleus as a transcriptional co-activator with T-cell factor/lymphoid enhancer factor (TCF/LEF) to induce transcription of Wnt target genes. **(C)** The PI3K/Akt/mTOR signaling pathway. Growth factors bind to a receptor tyrosine kinase (RTK), causing dimerization and autophosphorylation of the receptors. PI3K is recruited to the membrane and catalyzes the production of phosphatidylinositol-3, 4, 5-triphosphate (PIP_3_) from phosphatidylinositol-4, 5-bisphosphate (PIP_2_) which is negatively regulated by phosphatase and tensin homolog (PTEN). PIP_3_ activates Akt, a serine/threonine kinase that regulates numerous cell survival and cell cycle processes, including the inhibition of GSK3β and TSC1/2. The inhibition of TSC1/2 releases Rheb to activate mTOR complex 1 (mTORC1), comprised of mTOR and the regulatory associated protein of mTOR (Raptor). The upregulation of mTORC1 activates several important cellular processes including protein synthesis, proliferation, cell growth, and autophagy. During times of cellular metabolic stress, LKB1, a serine-threonine kinase, activates AMP-activated protein kinase (AMPK) which activates TSC2 and inhibits Raptor to downregulate mTORC1 signaling. This figure was constructed using information from several reviews (Shaw, 2009; Kaidanovich-Beilin and Woodgett, 2011; Porta et al., 2014; Martin-Orozco et al., 2019; Prossomariti et al., 2020).

Both the Wnt/β-catenin pathway and the phosphatidylinositol-3-kinase/Akt/mechanistic target of rapamycin complex 1 (PI3K/Akt/mTOR) pathway are upregulated in many cancers. The Wnt/β-catenin signaling pathway is essential for development and plays an important regulatory role in cell proliferation, polarity, and fate determination as well as for adult tissue maintenance (Logan and Nusse, 2004; MacDonald et al., 2009; Martin-Orozco et al., 2019). The PI3K/Akt/mTOR signaling pathway regulates many key cellular processes including proliferation, growth, metabolism, and survival (Jiang et al., 2020). Numerous studies have implicated crosstalk between these pathways as contributing to both tumorigenesis and therapeutic resistance in several cancer types including colorectal cancer, T-cell acute lymphoblastic leukemia, gastric cancer, and cervical cancer (Choi et al., 2019; Evangelisti et al., 2020; Guerra et al., 2020; Prossomariti et al., 2020). Oncogenic aberrations in these pathways are implicated in a wide spectrum of malignancies and are present in many syndromes associated with germline mutations in CPGs including FAP(*APC*), PJS (*LKB1)*; Cowden syndrome (*PTEN*), and TSC (*TSC1/2*) (Figure 3).

#### Familial Adenomatous Polyposis

Germline truncating mutations in the *APC* tumor suppressor gene are implicated in FAP, a cancer predisposition syndrome, as loss of *APC* transforms the Wnt/β-catenin pathway into a constitutively active state. APC normally regulates the stability of β-catenin and the loss of APC causes the accumulation of β-catenin, greatly increasing transcription of Wnt target genes and ultimately leading to cancer in many cases (Fodde et al., 2001; Figure 3). Mouse models with different truncating *Apc* mutations develop varying numbers of polyps in the small intestine, from 3 to 300 (Moser et al., 1990; Fodde et al., 1994; Oshima et al., 1995), while an *Apc* null mutant engineered with concurrent *Cdx2* mutations, a modifier gene that encodes a transcription factor important for intestinal differentiation, instead developed polyps in the colon which is more representative of human FAP (Aoki et al., 2003). These models show that mice with different truncating *APC* mutations develop tumors, although at different frequencies, and that modifier mutations influence polyp formation.

The first zebrafish *apc* mutant was created using ENU mutagenesis and contains a truncating mutation in the mutation cluster region (MCR; defined by mutations identified in human FAP tumors) (Hurlstone et al., 2003). Homozygous mutant embryos (*apc*^*MCR*^) exhibit a curved body type, impaired cardiac valve development, and do not survive past 96 hours post-fertilization (hpf). Cardiac valve defects were rescued by overexpression of APC or Dickkopf-1, a Wnt inhibitor. The heterozygous *apc*^*MCR*^ mutants developed intestinal adenomas that histologically resembled mammalian polyps with high levels of β-catenin in proliferating cells, as well as liver and intestinal tumors by 15 months following treatment with the carcinogen 7,12-dimethylbenz[a]anthracene (Haramis et al., 2006). LOH of *apc* was not detected in these lesions, which is normally found in human FAP tumors, but the accumulation of β-catenin was suggestive that APC functions were disrupted. A later study demonstrated that homozygous *apc*^*MCR*^ zebrafish embryos had a 34% reduction in intestinal epithelial cells and revealed that β-catenin was upregulated in these cells but was restricted to the cytoplasm (Phelps et al., 2009). Further, they showed that through a β-catenin-independent process, the *apc*^*MCR*^ embryos had suppressed intestinal cell differentiation associated with increased expression of *ctbp1*, a transcriptional co-repressor. Injection of oncogenic KRAS^*G12D*^ mRNA into *apc*^*MCR*^ embryos caused the nuclear accumulation of β-catenin, which greatly increased proliferation and resulted in a four-fold increase of the total intestinal cell numbers compared to non-injected *apc*^*MCR*^ embryos. This was not observed in KRAS^*G12D*^ injected wild-type embryos, suggesting that additional mutations may be required following the loss of *APC* to trigger the nuclear accumulation of β-catenin and robust proliferation. These characteristics are often observed in the malignant transformation of FAP adenomas as well as sporadic carcinomas (Phelps et al., 2009).

For years, the consensus was that APC mutations alone were responsible for the initiation of adenomas, but whole-genome sequencing of adenomas from FAP patients has implicated other non-Wnt genes (Delacruz et al., 2019). *CCR4-NOT Transcription Complex Subunit 3 (CNOT3)*, a gene important for regulating mRNA transcription, was identified as the most commonly mutated gene (excluding *APC*) in 20% of 37 FAP adenomas compared to only 1% in the sporadic/inherited colon cancer subset from the TGCA dataset (Gao J. et al., 2013; Delacruz et al., 2019). Two of the most frequent mutations identified were *CNOT3*^*E20K*^ and *CNOT3*^*E70K*^. Knockdown of *cnot3a* in *apc*^*MCR*^ zebrafish embryos decreased intestinal development and differentiation (Delacruz et al., 2019). Additionally, introducing mutated human *CNOT3*^*E20K*^ mRNA into *apc*^*MCR*^ embryos rescued intestinal differentiation while *CNOT3*^*E70K*^ mRNA did not, suggesting that E70K is an inactivating mutation. *CNOT3*^*E70K*^ is commonly found in adenoma tissues and a variety of cancers, but the discovery of its inhibitory effect on intestinal differentiation is novel and suggests that *CNOT3*^*E70K*^ may contribute to the progression of adenomas to carcinomas (Delacruz et al., 2019).

One main tumor-suppressive function of APC is to promote intestinal differentiation through regulation of the retinoic acid (RA) biosynthesis pathway (Jette et al., 2004; Nadauld et al., 2004). Zebrafish *apc*^*MCR*^ embryos demonstrate a lack of colonocyte differentiation which is rescued by treatment with RA (Nadauld et al., 2005). Further, *CYP26A1*, a gene encoding one of the major RA catabolic enzymes, is upregulated in human FAP adenomas and sporadic colon carcinomas that have truncating *APC* mutations as well as in the intestine of zebrafish *apc*^*MCR*^ embryos (Shelton et al., 2006). Inhibition of *cyp26a1* in *apc*^*MCR*^ zebrafish restored intestinal differentiation, further demonstrating the role of APC in RA biosynthesis regulation and implicates CYP26A1 as a potential therapeutic target. A follow-up study demonstrated that deficiencies in the RA biosynthesis pathway in *apc*^*MCR*^ embryos led to the upregulation of *cebpb*, a transcription factor gene, which was associated with increased cyclooxygenase-2 (cox-2) expression, also known as ptgs2a (prostaglandin-endoperoxide synthase 2a) (Eisinger et al., 2006). Elevated COX-2 expression is observed in colorectal carcinomas, is associated with the loss of APC, and ultimately drives the activation of Wnt target genes. Additionally, numerous genes important for intestinal cell fate determination are hypomethylated in homozygous *apc*^*MCR*^ embryos and this methylation loss was reversed upon treatment with RA (Rai et al., 2010). The hypomethylation of these key genes was associated with impaired differentiation, as intestinal cells were maintained in a progenitor-like state.

Given the role of the Wnt/β-catenin pathway in tumorigenesis, it has been a target for therapy development. Zebrafish embryos were exposed to 6-bromoindirubin-3′-oxime, a GSK3 inhibitor that activates Wnt signaling, and underwent a drug screen where axitinib, a clinically approved VEGF inhibitor, was identified as the strongest inhibitor of the Wnt pathway (Qu et al., 2016). Importantly, treatment of adult zebrafish with axitinib for 6 days revealed no observable defects or morphological changes at the concentration required to inhibit Wnt signaling. This provides preclinical evidence that axitinib could be repurposed as a cancer therapy. Further, cross regulation between the Wnt/β-catenin and the mTOR (previously known as zTOR in zebrafish) pathways has been demonstrated in the *apc*^*MCR*^ zebrafish mutants. *In vitro* studies have revealed that APC activates GSK3β which inhibits mTORC1 activity (Inoki et al., 2006; Valvezan et al., 2012). Consistent with this, mTORC1 activity is strongly upregulated in *apc*^*MCR*^ zebrafish mutants (Valvezan et al., 2014) and inhibition of mTORC1 partially rescues embryonic *apc*^*MCR*^ defects, with further rescue of curved body types by inhibiting both mTORC1 and Wnt signaling (Valvezan et al., 2014). This suggests that simultaneous targeting of these pathways is a potentially robust therapeutic strategy.

#### Peutz-Jeghers Syndrome

Peutz-Jeghers syndrome is a cancer predisposition disorder caused by germline inactivating mutations in *LKB1* (Hemminki et al., 1998). Affected individuals with PJS develop both benign hamartomas and early-onset cancers, especially in the gastrointestinal tract, and females with PJS have an increased risk of developing breast cancer (Giardiello et al., 1987; Hearle et al., 2006). Somatic *LKB1* mutations have been implicated in a spectrum of malignancies, including lung cancer, cervical cancer, breast cancer, malignant melanoma, non-small cell lung carcinomas, and acute myeloid leukemia (AML) (Guldberg et al., 1999; Fenton et al., 2006; Ji et al., 2007; Matsumoto et al., 2007; Wingo et al., 2009; Marinaccio et al., 2020). During periods of cellular metabolic stress, such as low levels of ATP, glucose, or oxygen, LKB1 plays a major role in energy metabolism control to maintain homeostasis (Kullmann and Krahn, 2018). Normally following a metabolic stress event, LKB1 activates AMP-activated protein kinase (AMPK), leading to a signaling cascade that inhibits the mTOR pathway (Figure 3C), thus preventing cell replication and growth in unfavorable conditions (Hardie, 2007; Kullmann and Krahn, 2018).

Zebrafish *lkb1*^–/–^ mutants exhibit a starvation phenotype that is lethal by 7–8 dpf (Van Der Velden et al., 2011). Following depletion of the yolk which occurs around 5–7 dpf, homozygous mutants develop an accelerated metabolism that results in rapid depletion of their energy reserves. Treatment with rapamycin, an mTOR inhibitor, slowed the metabolic rate and prolonged survival, but did not fully rescue the mutant phenotype. These mutants demonstrate that lkb1 is crucial for metabolic control during energetic stress, like yolk depletion, and represent a potential screening tool for compounds that decrease the accelerated metabolic rate, a common characteristic of cancer cells. The *lkb1* null mutants have also demonstrated that loss of *lkb1* prevents the activation of autophagy during the metabolic stress that ensues from the switch of nutrient absorption from the yolk to external nutrients (Mans et al., 2017). Autophagy is a cellular mechanism of self-digestion important for maintaining cellular homeostasis both under basal conditions and during metabolic stress, and defects in this process have been implicated in cancer (Rabinowitz and White, 2010). Impaired autophagy in the *lkb1* mutants was associated with an accumulation of p62, a multifunctional signaling protein important for mTORC1 activation and autophagy regulation (Duran et al., 2011), as well as decreased survival (Mans et al., 2017).

Additionally, zebrafish *lkb1*^–/–^ mutant embryos have glucose homeostasis defects, enhanced glycolysis, and increased lactate production (Kuang et al., 2016). This is similar to the metabolic state observed in cancer cells which often rely on aerobic glycolysis and fermentation of pyruvate to lactate to produce ATP instead of oxidative phosphorylation. This is termed the Warburg effect which, although inefficient for generating ATP, preserves carbon to support anabolic processes for increased proliferation (Vander Heiden et al., 2009). As a proof-of-principle, treatment with dichloroacetate, an aerobic glycolysis inhibitor, decreased lactate production in *lkb1*^–/–^ embryos, showing the potential of these mutants to be used as a drug screening platform to identify compounds that inhibit aerobic glycolysis (Kuang et al., 2016).

#### Cowden Syndrome

Cowden syndrome is a rare hamartoma tumor syndrome that is characterized by mutations in *PTEN* and is associated with an increased susceptibility to benign and malignant tumors including breast, skin, and thyroid cancers (Eng, 2003). *PTEN* is a tumor suppressor gene that is frequently inactivated in many cancers and normally functions as a negative regulator of the PI3K/Akt/mTOR signaling pathway (Sansal and Sellers, 2004; Figure 3C). *PTEN* has two homologs in zebrafish, *ptena* and *ptenb*, and while single homozygous mutants develop normally, double homozygous mutants do not survive past 5 dpf (Faucherre et al., 2008). The *ptenb^–/–^* single mutants developed spontaneous eye tumors, identified as neuroepitheliomas, around seven months of age. The presence of just one *pten* wild-type allele, as either *ptena*^–/^*^–^*; *ptenb*^+/^*^–^* or *ptena*^+/^*^–^*; *ptenb*^–/^*^–^*, is sufficient for survival to adulthood and the three mutant alleles are associated with spontaneous tumor growth (Faucherre et al., 2008; Choorapoikayil et al., 2012). Double homozygous *pten* embryos have increased proliferation and decreased apoptosis, and treatment with LY294002, a PI3K inhibitor, rescued all developmental phenotypes (Faucherre et al., 2008). Compared to other single and double mutants, the *ptena*^+/^*^–^*; *ptenb*^–/^*^–^* mutants were more susceptible to tumor development as they had a 10% incidence rate with 26/30 tumors found near the eye, while only 1/42 *ptena*^–/^*^–^*; *ptenb*^+/^*^–^* mutants developed a tumor (Choorapoikayil et al., 2012). The tumors were diagnosed as hemangiosarcomas, malignant hamartomas made up of vascular tissue, consistent with the increased frequency of hamartomas found in individuals with Cowden syndrome, and were characterized by increased proliferation, upregulated Akt signaling, and haploinsufficiency of either single *pten* allele (Choorapoikayil et al., 2012; Stumpf et al., 2015). A follow-up study from this group revealed that *ptena*^–/^*^–^*; *ptenb*^–/^*^–^* embryos have enhanced angiogenesis which was rescued by treatment with either LY294002 or sunitinib, a receptor tyrosine kinase (RTK) inhibitor used to reduce angiogenesis in cancer patients (Choorapoikayil et al., 2013). However, treatment with sunitinib greatly increased *vegfaa* expression in both the *ptena*^–/^*^–^*; *ptenb*^–/^*^–^* embryos and the hemangiosarcomas that developed in the adult haploinsufficient mutants (Choorapoikayil et al., 2013). *VEGFA*, the human ortholog of zebrafish *vegfaa*, encodes a ligand crucial for angiogenesis and thus its elevated expression may explain the relapse that is clinically observed following sunitinib treatment in some cases (Tonini et al., 2010; Kikuchi et al., 2012). Importantly, combination treatment with both LY294002 and sunitinib was successful in reducing neovascularization while maintaining normal levels of *vegfaa* in the *ptena*^–/^*^–^*; *ptenb*^–/^*^–^* embryos and thus has potential as a novel treatment strategy for the prevention of tumor angiogenesis (Choorapoikayil et al., 2013).

#### Tuberous Sclerosis Complex

Tuberous sclerosis complex is an autosomal dominant disorder associated with an increased susceptibility to renal cell carcinomas, as well as benign hamartomas that appear in multiple organs (Crino et al., 2006). This disorder is characterized by mutations in either the *TSC1* or *TSC2* CPGs which normally function to inhibit Rheb, a GTPase that activates the mTOR pathway (Figure 3C). As such, upregulation of the mTOR pathway is commonly observed in individuals with TSC (Zhang et al., 2003). Interestingly, *TSC* mutations are also associated with decreased cell proliferation and increased apoptosis which may counteract the effects of increased mTOR signaling enough to prevent the malignant transformation (Kim et al., 2013). Indeed, individuals with TSC mostly develop benign tumors compared to individuals with Cowden’s syndrome who also experience mTOR upregulation but develop multiple malignancies.

Morpholino knockdown of *tsc1a*, one zebrafish paralog of *TSC1*, led to kidney cyst formation, left-right asymmetry defects, increased ciliary length, and upregulation of the mTOR pathway (DiBella et al., 2009). Homozygous *tsc2* mutant embryos (*tsc2*^*vu242*^) had greatly increased mTORC1 signaling, consistent with TSC patients, but do not survive past 11 dpf (Kim et al., 2011). Heterozygous mutants had only a moderate increase of mTORC1 signaling and showed no development defects, suggesting there may be a minimum threshold of pathogenic mTORC1 signaling. As both *tsc1a* and *tsc2* homozygous mutations were embryonically lethal and heterozygous mutants did not develop tumors, Kim et al. (2013) introduced the heterozygous *tsc2*^*vu242*^ mutation into a mutant *tp53* background to investigate the tumorigenic potential of *tsc2* mutations. Compared to the *tp53*^*M214K/M214K*^ mutants, the *tsc2*^*vu242/*+^; *tp53*^*M214K/M214K*^ compound mutants had an increased rate of multiple malignancies, mTORC1 signaling, and angiogenesis while treatment with rapamycin caused regression of the tumor and tumor-associated blood vessels.

### RASopathies Associated With CPG Mutations

**FIGURE 4:**
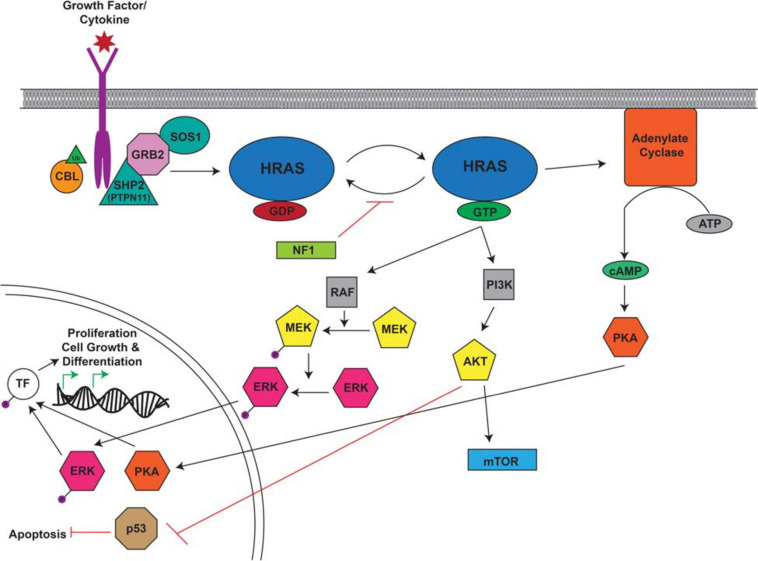
Overview of the RAS Signaling Pathway. The Ras signaling cascade is stimulated by the binding of a growth factor/cytokine to the appropriate RTK. C-CBL (Casitas B-lineage lymphoma) is an E3 ubiquitin ligase responsible for the ubiquitination of RTKs leading to their internalization and degradation. The activated receptor binds the adaptor proteins GRB2 (growth factor receptor bound protein 2) and SHP2/PTPN11 (protein tyrosine phosphatase non-receptor type 11) and recruits SOS1 (son of sevenless homolog 1). SOS1 facilitates the conversion of RAS from its inactive to active confirmation through the binding of GTP. RAS-GTP activates RAF, PI3K, and adenylate cyclase to cause a signaling cascade. RAF activates MEK which in turn activates ERK. Adenylate cyclase facilitates the production of cAMP which ultimately activates PKA. Activated ERK and PKA are protein kinases that translocate to the nucleus where they phosphorylate and activate transcription factors (TF) which drive the transcription of target genes involved in proliferation, cell growth, and differentiation. PI3K activates AKT which can activate mTOR and inhibit p53, leading to the inhibition of apoptosis. This figure was generated using information from several research articles and reviews (Ries et al., 2000; Kratz et al., 2006; Malaquias and Jorge, 2014; Wang et al., 2018).

RASopathies are a group of diseases caused by germline mutations in genes that regulate the Ras/MAPK pathway. Several RASopathies are associated with CPG mutations, including Noonan, LEOPARD, *CBL*-mutated, and Costello syndromes as well as neurofibromatosis type 1 (NF1). The Ras/Raf/Mek/Erk (MAPK) pathway is a key signaling cascade that promotes cell growth, division and differentiation (Molina and Adjei, 2006; Figure 4). Ras mutations and aberrant Ras signaling have been extensively studied in the context of malignancy as mutated Ras is found in about 20% of cancers (Prior et al., 2012). Patients with inherited or *de novo* congenital RASopathies are prone to variety of malignancies during childhood as well as later in life through second hit events that eliminate their remaining wild-type allele. Affected individuals also suffer from a variety of developmental phenotypes affecting multiple organs and tissue types which often leads to the initial RASopathy diagnosis.

#### Noonan/LEOPARD Syndrome

Noonan syndrome (NS) and LEOPARD syndrome (LS) are RASopathies that are typically grouped together as they are genotypically and phenotypically similar (Tartaglia et al., 2011; Martínez-Quintana and Rodríguez-González, 2013) and both syndromes predispose to malignancies such as AML (Jongmans et al., 2011). NS is characterized by growth retardation, skeletal deformities, facial dysmorphia and congenital heart defects, among other abnormalities (Tartaglia et al., 2001) and shares many overlapping features with LS which is defined by multiple lentigines, electrocardiographic abnormalities, ocular hypertelorism, pulmonary stenosis, abnormal genitalia, retardation of growth, and sensorineural deafness (Tartaglia et al., 2011).

Noonan syndrome and LS are heterogenous autosomal dominant diseases caused by mutations in several genes, including the CPG, *PTPN11*. *PTPN11* encodes the ubiquitously expressed Src homology region 2 domain containing phosphatase-2 (SHP2), a protein tyrosine phosphatase involved in relaying growth signals and activating RAS and the MAPK/ERK pathway (Neel et al., 2003; Figure 4). *PTPN11* is essential for many developmental pathways and is frequently deregulated in several types of cancer (Jongmans et al., 2011). Although phenotypically similar, *PTPN11* mutations in LS and NS have biologically distinct functions. GOF *PTPN11* mutations are found in roughly half of NS patients typically as missense mutations that activate PTPN11, while *PTPN11* mutations found in LS mainly affect catalytic residues in the PTP domain that suppress phosphatase activity (Tartaglia et al., 2011). Studies of NS and LS in zebrafish have focused on examining the underlying disease mechanisms of *ptpn11* deregulation and exploring the ways in which overlapping syndromic features can be caused by opposing effects on *ptpn11* catalytic activity. One such explanation implicates the shared activation of RAS/MAPK signaling in overlapping symptoms whereas distinct NS and LS features are the result of differences in PI3K/Akt pathway expression which is uniquely upregulated in LS patients and mouse models (Kontaridis et al., 2006; Serra-Nedelec et al., 2012).

The first of these studies overexpressed either NS or LS mutant mRNA, which most notably resulted in shorter zebrafish larvae that were affected by heart abnormalities and a ‘hammer-head’ like phenotype reminiscent of both patients and mouse models (Jopling et al., 2007). Furthermore, the simultaneous overexpression of NS and LS mutant mRNA did not increase the severity of the phenotypes; a finding consistent with the established opposing effects of the two mutations. Similar phenotypes have been observed in subsequent studies using morpholinos to knockdown endogenous expression of zebrafish *PTPN11* orthologues, *ptpn11a* and *ptpn11b*, injecting NS or LS mutant mRNA, and in *ptpn11a^–/–^* and *ptpn11a*^–/^*^–^*; *ptpn11b*^–/^*^–^* mutant fish (Stewart et al., 2010; Bonetti et al., 2014a, b). In contrast to homozygous knockout mice that die pre-implantation, zebrafish *ptpn11a*^–/^*^–^* and *ptpn11a*^–/^*^–^*; *ptpn11b*^–/^*^–^* mutants live to 5dpf, while *ptpn11b^–/–^* mutants survive to adulthood and are fertile (Yang et al., 2006; Bonetti et al., 2014b). Together, mortality and expression data, which demonstrated lack of significant *ptpn11b* expression during early embryogenesis, implicate *ptpn11a* as the more influential ortholog (Bonetti et al., 2014b). Decreased expression of *erk* was observed in morphant and double homozygous knockout models while PI3K signaling remained unaffected (Stewart et al., 2010; Bonetti et al., 2014b). Congruent with previous observations, phospho-Akt levels increased only in embryos injected with LS mutant mRNA (Bonetti et al., 2014a). Additionally, NS/LS zebrafish models have been used to investigate the role of *PTPN11* mutations in both congenital sensorineural hearing loss and heart defects which are found in individuals with NS and LS (Sarkozy et al., 2008; Bonetti et al., 2014a; Gao et al., 2020).

#### c-CBL Mutation Associated Syndrome

Clinical features of patients with germline c-*CBL (Casitas B-lineage Lymphoma)* mutations partially overlap those of NS, including developmental delays, growth defects, facial dysmorphia, and a predisposition to hematological malignancies, specifically juvenile myelomonocytic leukemia (JMML) (Niemeyer et al., 2010; Perez et al., 2010). CBL is an E3 ubiquitin ligase that targets both active β-catenin and FLT3 (fms related receptor tyrosine kinase 3): two factors important for tumor-induced angiogenesis which have also been implicated in AML (Lyle et al., 2019).

The *c-cbl*^*H382Y*^ zebrafish ENU-induced line (Peng et al., 2015) harbors a missense mutation within a highly conserved domain critical for transfer of ubiquitin to target molecules (Thien and Langdon, 2001; Deshaies and Joazeiro, 2009). *c-cbl*^*H382Y*^ zebrafish displayed a myeloproliferative phenotype with increased numbers of hematopoietic stem cells (HSCs), erythrocytes and neutrophils at 5 dpf and a median survival time of only 15 dpf (Peng et al., 2015). phospho-S10 histone H3 (pH3) antibody/staining revealed that these HSCs were highly proliferative and remained more abundant in older fish, despite no obvious impairments in HSC differentiation. In contrast to the *c-cbl*^*H382Y*^ line, zebrafish injected with human phospho-tyrosine-inactive mutation *c-CBL*^*Y731F*^ mRNA, an amino acid residue that when phosphorylated suppresses Wnt signalling, had enhanced angiogenesis and increased expression *vegfa* while those injected with the phosphomimetic mutation *c-CBL*^*Y731E*^ mRNA displayed impaired angiogenesis and decreased expression *vegfa* (Shivanna et al., 2015). Thus, the different phenotypes were likely observed as the different missense mutations affect the binding of c-cbl to different proteins.

#### Costello Syndrome

Costello syndrome (CS), another NS-like syndrome, is caused by inherited activating mutations in *HRAS*, typically in the form of missense mutations (Aoki et al., 2005). CS has phenotypic features similar to NS, as well as the distinctive and common formation of benign cutaneous papillomata within the perinasal and perianal regions (Aoki et al., 2005). Santoriello and colleagues developed a zebrafish model of CS that ubiquitously expresses the constituently active *HRAS*^*G12V*^ gene (Santoriello et al., 2009). *HRAS*^*G12V*^ transgenic fish were shorter, had a smaller heart with thicker walls, a flattened head and suffered from scoliosis; features indicative of Costello syndrome. Tumors frequently formed in fish between 5–12 months and included melanoma, gastrointestinal carcinoma, hepatocarcinoma and rhabdomyosarcoma. Congruent with mouse models (Schuhmacher et al., 2008), ERK1/2 and Akt phosphorylation were not upregulated in HRAS^*G12V*^ transgenic zebrafish and remain unlikely drivers of CS pathology; rather, the ability of HRAS to induce hyperproliferation, DNA damage, and cellular senescence through the DNA damage response (DDR) is a well-documented cause (Di Micco et al., 2006). Indeed, HRAS^*G12V*^ transgenic zebrafish demonstrated oncogene-induced senescence in regions of the heart and brain as well as increased expression of DDR markers within various tissues (Santoriello et al., 2009).

#### Neurofibromatosis Type 1

Neurofibromatosis type 1 is an autosomal dominant disorder caused by mutations to the RAS GTPase activating domain of the *NF1* gene. This mutation renders NF1 unable to hydrolyze RAS-bound GTP to GDP, ultimately activating the RAS signaling pathway (Cichowski and Jacks, 2001; Figure 4). Affected individuals develop cardiovascular abnormalities, nervous system defects including cognitive defects, and an increased incidence of malignancies, including JMML, gliomas, and MPNSTs. Homozygous deletion of murine *Nf1* results in embryonic lethality mid-gestation while heterozygous mice remain viable and fertile, but this mutant does not fully recapitulate the human phenotype (Brannan et al., 1994; Silva et al., 1997). More complex mouse models that use Cre/lox technology to express mutant *NF1* within specific cell populations better reproduce specific features of NF1 but lack the ability to study the disease as a whole (Zhu, 2002; Gitler et al., 2003).

Zebrafish have been successfully used to widen the scope of *in vivo* NF1 research using ZFN-mediated knockout of *NF1* paralogues, *nf1a* and *nf1b*, and allogenic transplantation techniques. Double homozygous *nf1* mutant zebrafish display macrocephaly as well as an increase in oligodendrocyte precursor cell (OPC) numbers and migration within the spinal cord (Shin et al., 2012). Further, defects in myelin structure formation and axon wrapping lead to decreased myelination in double homozygous *nf1* mutant fish, despite no change in *myb* (*myelin basic protein*) expression within the central nervous system. Several studies have postulated *NF1* as a positive regulator of adenylate cyclase (Guo et al., 2000; Tong et al., 2002; Dasgupta et al., 2003) and linked cAMP signaling to the expression of myelin components. *NF1* mutations associated with cognitive dysfunction have been linked to regions outside of the GTPase-activating protein-related domain (GRD) (Fahsold et al., 2000), suggesting that some features of NF1 may be caused by aberrant cAMP signaling rather than activated Ras. The memory formation of *nf1* mutant larvae was tested by evaluating the O-bend response to light/dark stimulus (Wolman et al., 2014). Trained *nf1* mutant larvae had a delayed O-bend response which was improved upon treatment with chemical stimulants for the cAMP pathway, but not MAPK or PI3K inhibitors. Similarly, when testing memory recall, *nf1* mutants again showed deficits, however these fish showed improvements over the course of the experiment, suggesting that cognitive defects are reversible and that patients may benefit from specialized learning environments and cAMP pathway effectors. Additionally, when crossed into a p53 null background, *nf1* mutants develop high-grade gliomas and MPNSTs, similar to what is commonly observed in NF1 patients (Shin et al., 2012).

Neurofibromatosis type 1 modulates neuronal differentiation (Hegedus et al., 2007), and NF1 patients frequently develop benign neurofibromas that can undergo malignant transformation to MPNSTs, commonly through the loss of CDKN2A and p53 as well as amplification of *PDGFRA* (platelet-derived growth factor receptor A) (Mantripragada et al., 2008; Zietsch et al., 2010; Lee et al., 2014). The over-expression of *PDGFRA* has been linked to the malignant transformation of MPNSTs (Holtkamp et al., 2004). When modeled in transgenic zebrafish, overexpressing *PDGFRA* in a *nf1a*^+/–^; *nf1b*^–/–^; *tp53*^*M214K/M214K*^ background accelerated the onset of MPNSTs (Ki et al., 2017). Interestingly, over-expression of wild-type *PDGFRA*, was better at accelerating tumor development than the constitutively active *PDGFRA* which activated Ras signaling to levels higher than optimal for tumor growth. MPNST tumors from wild-type *PDGFRA* transgenic fish were harvested and transplanted into *nf1a*^+/–^; *nf1b*^–/–^; *tp53*^*M214K/M214K*^ larvae, establishing an allogenic transplant model that can rapidly test effectiveness of therapies. Indeed, sunitinib treatment effectively arrested progression of the transplanted MPNSTs in these mutants, and treatment with both sunitinib and trametinib, a MEK inhibitor, enhanced this therapeutic effect. Additionally, both DNA topoisomerase I (Topo I) targeting drugs and mTOR kinase inhibitors were identified as the most effective single agent therapies that avoided excessive toxicity (Ki et al., 2019). Treating with Topo I inhibitor Irinotecan and mTOR inhibitor AZD2014, acted synergistically to induce apoptosis and block protein synthesis resulting in tumor cell death.

### Hematopoietic Disorders Associated With CPG Mutations

**FIGURE 5:**
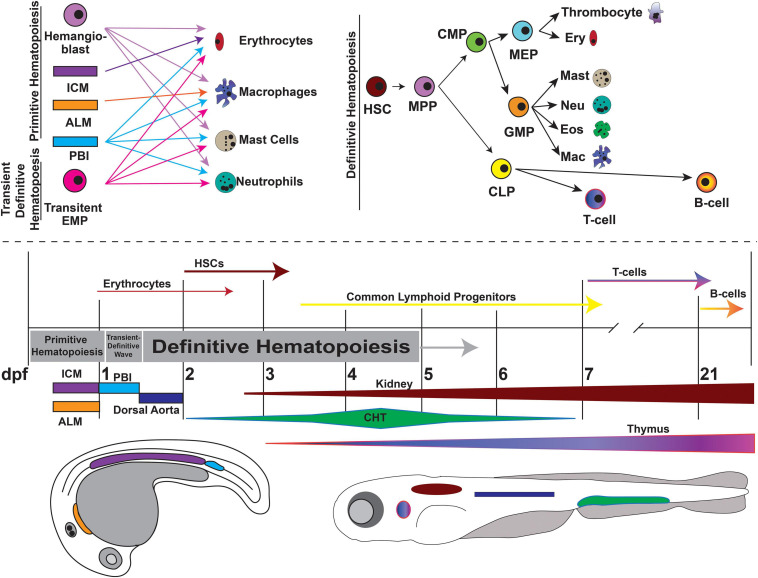
Overview of zebrafish hematopoiesis. Zebrafish hematopoiesis occurs in two waves, primitive and definitive, the latter of which recapitulates all the same blood cell types as humans. During the primitive wave, hemangioblasts give rise to a portion of primitive erythrocytes and granulocytes. The main source of primitive granulocyte production occurs within the anterior lateral mesoderm (ALM) (orange), while primitive erythrocyte productions occurs in the intermediate cell mass (ICM) (purple). Once circulation begins at approximately 24 hpf, primitive blood production transitions to the posterior blood island (PBI) (blue). Between 24 and 36 hpf a transient wave occurs in which transient bipotential erythromyeloid progenitor cells (EMPs). By 36 hpf, the first hematopoietic stem cells (HSCs) arise from the ventral wall of the dorsal aorta (navy), a site analogous to the aorta-gonad-mesonephros (AGM) in mammals. Once HSCs arise, they migrate to the caudal hematopoietic tissue (CHT) (previously the PBI), a structure analogous to the mammalian fetal liver. It is in the CHT that definitive erythroid and myeloid cells are produced and replace their primitive counterparts. By 4dpf HSCs migrate to and seed the kidney marrow (dark red), the equivalent of bone marrow in mammals. The first T-cells originate at approximately 7 dpf and mature in the thymus (purple/pink), while B-cells are not present until 21 dpf. This figure was constructed using information from published reviews (Vogeli et al., 2006; Rasighaemi et al., 2015; Gore et al., 2018). (MPP, Multipotent Progenitor; CLP, Common Lymphoid Progenitor; CMP, Common Myeloid Progenitor; MEP, Megakaryocyte-Erythroid Progenitor; GMP, Granulocyte-Monocyte Progenitor).

Several familial hematologic syndromes are associated with mutations in CPGs. Inherited bone marrow failure syndromes (IBMFS), including dyskeratosis congenita (DC), Schwachman-Diamond syndrome (SDS) and Fanconi anemia (FA) (discussed in a later section) are rare heterogeneous disorders caused by genetic defects that impair normal hematopoiesis, leading to anemia, cytopenia, and a variety of solid tissues/organ abnormalities. Additionally, these disorders predispose patients to develop malignancies. Zebrafish have long been used to study blood development and model hematologic diseases due to their high level of genetic conservation, especially within gene pathways critical to hematopoiesis (Jing and Zon, 2011). Similar to humans, zebrafish blood development occurs in two waves and within analogous structures recapitulating all blood cell types (Figure 5) (Davidson and Zon, 2004).

#### Dyskeratosis Congenita

Dyskeratosis congenita is a heterogenous disease caused by mutations in multiple genes involved with telomere maintenance including telomerase reverse transcriptase (*TERT*) and members of the H/ACA ribonucleoprotein (RNP) complex. H/ACA RNPs, NOP10 and DKC1 (dyskerin pseudouridine synthase 1) are involved in ribosome biogenesis, splicing and processing of various RNAs, and are also part of the telomerase complex together with TERT and the telomerase RNA component (Kiss et al., 2010). Telomerase is important for counteracting the progressive shortening of replicated DNA, especially in highly proliferative cell types and stem cells, and prevents activation of DDR pathways that could result in cellular senescence or apoptosis. Affected individuals develop nail dystrophy, mucosal leukoplakia, skin hyperpigmentation, increased risk of both hematological and solid cancers as well as bone marrow failure (BMF) (Cole et al., 1930; Alter et al., 2009). DC mortality is predominantly due to BMF, although the cumulative incidence of cancer is high with roughly 40% of patients developing a malignancy by the age of 50 (Alter et al., 2009). Mouse models of DC are limited because mouse telomeres are roughly 5–10 times longer than their human counterparts (10–15 kb) (Kipling and Cooke, 1990) while zebrafish telomeres are more similar to humans at 15–20 kb (Carneiro et al., 2016).

Zebrafish represent an advantageous platform for modeling DC and telomere deficiencies, and *tert* mutant zebrafish have reduced telomere length, premature aging, decreased fertility, and shorter lifespans (Anchelin et al., 2013). Dramatic telomere shortening triggered p53 activation, whereas p53 knockdown rescued apoptosis in larvae although telomere length remained short. Adult *tert* mutants had increased levels of apoptosis within the kidney marrow (the equivalent of human bone marrow), which also showed signs of depletion upon histopathological analysis (Henriques et al., 2013). Notably, reintroducing *tert* into second-generation mutants which typically died by 7dpf prevented both mortality and further telomere shortening (Anchelin et al., 2013). This suggests that drugs that activate wild-type *TERT* expression could be a potential therapy for DC.

The most severe form of DC, Hoyeraal-Hreidarsson syndrome, is caused by mutations to the catalytic domain of *dyskerin (DKC1*), suggesting that telomere-independent mechanisms contribute to DC (Knight et al., 1999; Zhang et al., 2012). Morpholino-mediated knockdown of zebrafish *dkc1* significantly reduced the development of all blood cell lineages during definitive hematopoiesis (Zhang et al., 2012). Similar to the knockdown of *tert, dkc1* morphants had a significant increase in the expression of p53 and pro-apoptotic genes such as *bax1.* Hematopoietic defects could be reversed by inhibiting p53 expression in *dkc1* morphants, again suggesting that DC-associated cytopenia is partially p53 dependent. Interestingly, investigators observed significant defects in 18S rRNA production prior to the onset of abnormal blood development in *dkc1I* morphants and *nop10* mutant fish (Pereboom et al., 2011; Zhang et al., 2012). Extensive p53-mediated apoptosis in *nop10* mutants was linked to increased binding of Mdm2 to Rps7 resulting in degradation of Mdm2 protein which is normally responsible for p53 degradation. Furthermore, *nop10* mutants failed to form HSCs despite no evidence of telomere shortening, and cytopenia was rescued through the inactivation of p53, providing additional evidence that p53-mediated apoptosis of HSCs is driven by ribosomal biogenesis defects and not solely by telomere shortening (Pereboom et al., 2011).

#### Shwachman-Diamond Syndrome

Schwachman-Diamond syndrome is an autosomal recessive disorder caused by mutations in *SBDS* that either interrupt transcript splice sites or result in a truncated protein (Boocock et al., 2003). Affected individuals develop exocrine pancreatic insufficiency, impaired hematopoiesis, skeletal abnormalities, gastrointestinal dysfunction and an increased risk of leukemia with ∼36% of patients developing myelodysplastic syndrome (MDS) (a syndrome which represents a pre-leukemia state) or AML by age 30 (Burroughs et al., 2009; McReynolds and Savage, 2017). SBDS is involved in ribosome biogenesis and function with a role in 40S and 60S ribosomal subunit maturation and assembly (Stepensky et al., 2017; Warren, 2018; Tan et al., 2019). Zebrafish *sbds* shares 86% amino acid identity with humans and retains the same intron/exon boundaries, making it an advantageous model for SDS modelling (Oyarbide et al., 2019). Morpholino-mediated knockdown of *sbds* significantly decreases neutrophil proliferation and migration as well as results in pancreatic hypoplasia and abnormal morphology (Venkatasubramani and Mayer, 2008; Provost et al., 2012). Additionally, *sbds* morphant embryos displayed broad changes in ribosomal gene expression. Simultaneous knockdown of p53 did not restore normal neutrophil numbers or normal pancreatic morphology, suggesting SDS pathology is not p53 dependent. Two CRISPR/Cas9 generated *sbds* mutant zebrafish, *sbds*^*nu167*^ producing a truncated protein and *sbds*^*nu132*^ with a 7 amino acid in-frame deletion, had reduced length and weight and died by 21 dpf, which was not rescued by crossing *sbds* mutants into a *tp53* mutant (*p53*^*M214K/M214K*^) background (Oyarbide et al., 2020). Similar to the morphant studies, *sbds*^*nu132*^ displayed neutropenia, while the number of macrophages and hemoglobin production remained normal. In addition, kidney marrow morphology remained normal at 21 dpf. In contrast to morphant studies, neither pancreatic hypoplasia nor changes to *ptf1a* expression were observed in *sbds* mutant larvae up to 15 dpf but rather pancreatic atrophy began in 15–21 dpf mutant fish after seemingly normal development. Therefore, zebrafish *sbds* models recapitulate several patient phenotypes including neutropenia and pancreatic defects, although the mechanisms that lead to pancreatic atrophy have not yet been explained.

#### Wiskott-Aldrich Syndrome and X-Linked Congenital Neutropenia

Wiskott-Aldrich syndrome (WAS) and X-linked congenital neutropenia (XLN) are severe immunodeficiency diseases caused by both LOF and GOF mutations in the Wiskott-Aldrich syndrome protein (WASp), respectively. WASp is an important regulator of the actin cytoskeleton in hematopoietic cells and is therefore a key player in leukocyte motility, phagocytosis, and a productive immune response (Tsuboi and Meerloo, 2007; Thrasher and Burns, 2010). Individuals with WAS or XLN develop frequent and recurrent infections while WAS patients also suffer from autoimmunity, bleeding disorders, and various types of leukemia and lymphoma at an incidence of roughly 13–22%, typically before 18 years of age (Sullivan et al., 1994; Imai et al., 2004; Keszei et al., 2018). One explanation for such predisposition is a failure to identify and eradicate malignant cells prior to tumor formation due to compromised immune function. Zebrafish offer a unique advantage over mouse models as the optical clarity of zebrafish larvae allows for the live imaging and analysis of immune cell response and migration.

Both zebrafish homologs of *WASp*, *wasp1* (*waspb*) and *wasp2* (*waspa*), are well conserved with 52% and 41% amino acid similarity to humans, respectively, and are hematopoietically restricted (Cvejic et al., 2008). Live imaging of *lyz:eGFP* transgenic zebrafish (fluorescently labelled neutrophils and macrophages) with knockdown of *wasp1* revealed a reduction in neutrophils and macrophage migration to wounds in morphant larvae, while the number of immune cells remained normal. Furthermore, *wasp* morphants had decreased survival when inoculated with pneumococci (Rounioja et al., 2012), resembling life-threatening pneumococcal infections seen in individuals with WAS (Sullivan et al., 1994). Homozygous *wasp1* mutant zebrafish recapitulated the ineffective immune responses to both inflammatory wounds and bacterial challenges seen in morphant studies and further revealed that that the ineffective formation, maintenance and retraction of neutrophil pseudopods and uropods contributed to the defective immune response (Jones et al., 2013). Expression of *wasp* was replaced in the *wasp1* null background using a variety of patient-specific *WASp* transgenes and the expression of wild-type human WASp rescued neutrophil migration and wound recruitment and restored phagocytosis when challenged with *S. aureus*. In comparison, phospho-dead WASp^*Y291F*^ provided minimal improvement to neutrophil migration and did not improve microbe uptake by macrophages. Moreover, expression of constitutively active WASp^*I294T*^, observed in patients with XLN, resulted in an overall reduction of neutrophils but upon wound-response, WASp^*I294T*^ expressing neutrophils had significantly increased cell velocity (Jones et al., 2013).

#### GATA2 Deficiency Syndromes

GATA2 is a transcription factor critical to both primitive and definitive blood development and lymphatic vessel formation. *GATA2* haploinsufficiency, from inherited or spontaneous heterozygous mutations, produces a range of hematopoietic disorders in humans including monocytopenia, mycobacterium avium complex (MonoMAC) syndrome; dendritic cell, monocyte, B and NK lymphoid (DCML) deficiency; congenital neutropenia; and familial MDS/AML (Bigley and Collin, 2011; Dickinson et al., 2011; Hahn et al., 2011; Hsu et al., 2011). Affected individuals often present with infection, cytopenias, aplastic anemia and hypocellular bone marrow, of whom 75% will develop MDS/AML with a median age of onset of 20 years (Wlodarski et al., 2016, 2017). Control of Gata2 expression has been linked to a conserved +9.5 kb enhancer region (Hsu et al., 2013), where deletions are found in ∼10% of patients with MonoMAC syndrome (Wlodarski et al., 2016). Zebrafish have two highly conserved homologs of *GATA2*, *gata2a* and *gata2b*, with 57% and 67% conserved amino acid identity (Jin et al., 2004; Lutskiy et al., 2005; Gillis et al., 2009; Butko et al., 2015). Comprehensive expression and lineage tracing analysis indicates *gata2a* and *gata2b* zebrafish have distinct patterns of expression; *gata2a* is expressed throughout the endothelium and is more broadly required for vascular morphogenesis while *gata2b* is only present within the hemogenic endothelium of the dorsal aorta and has a more specialized role to activate *runx1* expression required for HSC formation (Butko et al., 2015). Thus, this study suggests segregated endothelial and hematopoietic functions of the two zebrafish *gata2* paralogs.

Using CRISPR/Cas9, Dobrzycki and colleagues introduced a deletion to the conserved +9.5 enhancer within intron 4 of zebrafish *gata2a* (*gata2a^Δ*i4/*Δ*i4*^*) (Dobrzycki et al., 2020). Unlike mouse Gata2^Δ+9.5^ models (Gao X. et al., 2013), *gata2a^Δ*i4/*Δ*i4*^* fish survive past embryogenesis, and had decreased expression of HSC markers, *runx1* and *cmyb*, during primitive hematopoiesis. Despite the recovery of *runx1* and *cmyb* expression to wild-type levels by 48hpf, adult *gata2a^Δ*i4/*Δ*i4*^* fish had half the number of blood cells within their kidney marrow and a surplus of immature blasts was found in 10% mutants. Additionally, these fish had a high incidence of cardiac edema and infection within less than 6 months of age, suggestive of the immune deficiency and lymphatic defects seen in MonoMAC syndrome (Dobrzycki et al., 2020).

Preliminary results from two *gata2b* mutant zebrafish models highlight how zebrafish can elucidate the link between GATA2 deficiencies and MDS/AML predisposition as *gata2b^+/–^* mutants have reduced myeloid differentiation and dysplastic myeloid cells in older animals (Avagyan et al., 2017; Gioacchino et al., 2019). Cre/Lox fluorescent lineage tracing technology is being used to assess the expansion of single-color clones within the kidney marrow of 3dpf *gata2b^+/–^* larvae with/without concurrent mutations (Avagyan et al., 2017). Juvenile *gata2b^+/–^* zebrafish had a decreased myeloid compartment in the kidney marrow of which 30% also had an expansion of single-color clones by 3 months post-fertilization, an event not observed in wild-type fish. This myelocytopenic phenotype is more profound in *gata2b^+/–^* fish when challenged with similar concurrent mutations, as compared to wild-type, and is indicative of a predisposition to MDS/AML.

#### Familial AML Associated With *CEBPA* Mutations

CEBPA, a transcription factor important for normal myelopoiesis and granulocyte differentiation, is mutated in ∼10% of individuals with AML (Pabst et al., 2001; Gombart et al., 2002). Of these, about half have biallelic mutations consisting of mutations in both hotspot N- and C- terminal regions that are typically acquired somatically. Mutations in the N-terminal region frequently produce frameshifts that lead to premature stop codons and translation of a dominant negative proteins, while C-terminal mutations are usually in-frame indels (Pabst et al., 2001; Nerlov, 2004). Germline mutations typically occur at the N-terminal hotspot and these individuals acquire a second C-terminal mutation somatically (Fuchs et al., 2008; Tawana et al., 2015). Although a rare occurrence, individuals with germline *CEBPA* mutations develop AML much earlier than sporadic AML at a median age of 25 versus 67 years (McReynolds and Savage, 2017).

Zebrafish studies reveal a conserved role for *cebpa* and its involvement in primitive erythropoiesis and HSC formation (Liu et al., 2007; Yuan et al., 2011, 2015). Myeloid defects, including a lack of embryonic neutrophils and macrophages were observed in three different *cebpa* mutant lines (*moli^*hkz7*^, cebpa^*sum2*^, cebpa^*sum3*^)* (Dai et al., 2016). Myeloid defects in *cebpa* deficient *moli*^*hkz7*^ mutants could be explained, in part, by the aberrant cell cycle arrest of myeloid progenitors once the definitive wave of hematopoiesis began, suggesting that *cebpa* is more important for myeloid progenitor migration and maintenance than initiation. To more accurately model *CEBPA*-associated MDS/AML, two new *cebpa* mutant zebrafish lines were engineered with either N- or C- terminal mutations (Hockings et al., 2018). Preliminary evidence demonstrates striking defects in mature myelocytes and monocytes in all double mutant larvae, while leukemic transformation was only observed in mutants between 4-6 weeks of age that carried at least one N-terminal mutation.

#### Familial Platelet Disorder With Predisposition to AML

Patients with familial platelet disorder with predisposition to AML (FPD/AML) suffer from thrombocytopenia, platelet dysfunction, increased risk of bleeding and of developing hematological malignancies, as 35–40% will develop MDS/AML by 33 years of age (Sood et al., 2017). FPD/AML is associated with germline mutations in *RUNX1*, a master transcription factor regulator of hematopoiesis and blood vessel development, most commonly small indels or point mutations that can lead to haploinsufficiency or dominant negative effects (Latger-Cannard et al., 2016). Monoallelic *RUNX1* mutations alone are insufficient to initiation leukemogenesis, rather secondary somatic mutations are required within *RUNX1* or other AML-associated genes (Song et al., 1999; Preudhomme et al., 2009; Ripperger et al., 2009; Schmit et al., 2015; Antony-Debré et al., 2016). Neither heterozygous *RUNX1* murine or zebrafish models develop FPD phenotypes, however a germline homozygous *Runx1* deletion is embryonically lethal in mice (Okuda et al., 1996) while zebrafish *runx1*^*W84X/W84X*^ mutants are viable to adulthood (Jin et al., 2012). Like human patients, homozygous *runx1*^*W84X*^ larvae have a severe reduction in both immature and mature neutrophils as well as in mature thrombocytes (Sood et al., 2010; Jin et al., 2012), mimicking findings in conditional knockout mice (Ichikawa et al., 2004; Growney et al., 2005). Furthermore, adult *runx1*^*W84X/W84X*^ fish display reduced number of B-cells (Chi et al., 2018), a phenotype reminiscent of common variable immunodeficiency disorder (Li et al., 2015). Critically, neither myelocyte dysplasia nor an MDS-like phenotype have been reported in *runx1*^*W84X/W84X*^ fish and it is likely that additional models involving second-hit mutations are needed to more accurately model FPD/AML in zebrafish (Chi et al., 2018).

#### Congenital Amegakaryocytic Thrombocytopenia

Congenital amegakaryocytic thrombocytopenia (CAMT) is a rare IBMFS that often presents in infancy with mutations in the gene *MPL* which encodes the receptor for thrombopoietin. Affected individuals develop severe thrombocytopenia, aplastic anemia, hemorrhage and a predisposition to leukemia (Germeshausen et al., 2006; Al-Qahtani, 2010). To date, only one CAMT zebrafish model exists as *mpl*^*smu3*^, a LOF mutant that experiences severe thrombocytopenia during embryonic development (Lin et al., 2017). Although the development of HSC and progenitor populations are unaffected, there was a significant reduction in thrombocyte precursor proliferation and thrombocyte markers remained significantly low in adult fish. Similar to the high risk of prolonged bleeding observed in CAMT, *mpl*^*smu3*^ larvae displayed reduced hemostasis and abnormal bleeding. To further adapt their model as a platform for high-throughput drug screening, Lin et al. generated the thrombocyte-specific fluorescent transgenic reporter line Tg(*mpl:eGFP)smu4; mpl^*smu3*^*, and as proof-of-principle, showed that treatment with recombinant human IL-11, a cytokine important for megakaryopoiesis (Peeters et al., 2008), modestly increased thrombocyte populations in *mpl*^*smu3*^ larvae.

### CPG Mutations Involved in DNA Repair and Genome Stability Maintenance

**FIGURE 6:**
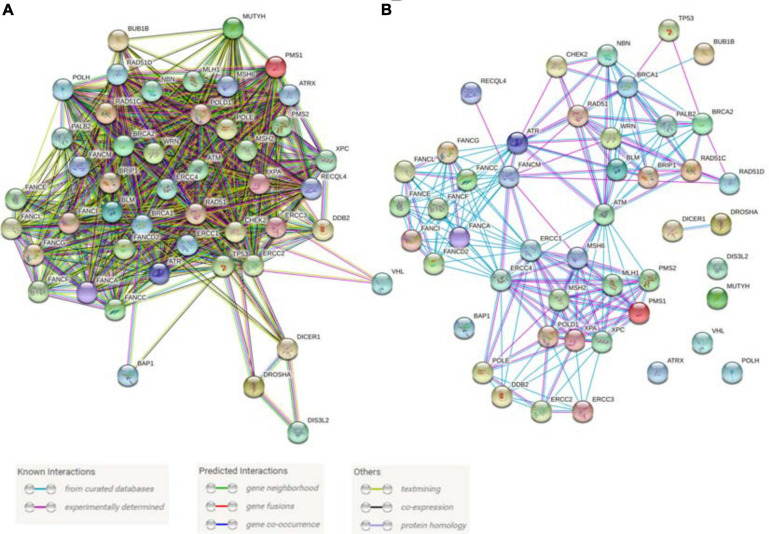
Predicted and known interactions among the cancer predisposition genes involved in DNA repair and genome stability maintenance. **(A)** All known interactions produce a very dense network. **(B)** A physical network with only experimentally verified results and curated database interactions were included. Both panels of the figure were produced using STRING database online tool (https://string-db.org/).

Genes linked to DNA repair and genome stability comprise the single most numerous group of CPGs and represent 50 out of 152 genes currently identified, further emphasizing the fundamental importance of genome stability maintenance in cancer development (Table 1). We undertook an analysis of the interactions within this group of 50 genes using the STRING database (Szklarczyk et al., 2015). The inclusion of all known associations results in a highly dense interaction network with only the DICER1-DROSHA-DIS3L2 group, VHL, and BAP1 exhibiting minimal links (Figure 6A) while focusing on well-established physical interactions results in a sparser network highlighting the best-known interactions (Figure 6B). Further, these 50 proteins are part of many dynamic protein complexes with several hundred members, but their discussion is beyond the scope of this review. Instead, we will focus on the contributions that zebrafish model research has made towards understanding the fundamental biology and cancer models associated with these CPGs.

#### Ataxia-Telangiectasia (A-T) Genes (*ATM*, *ATR* and *CHEK2*)

**FIGURE 7:**
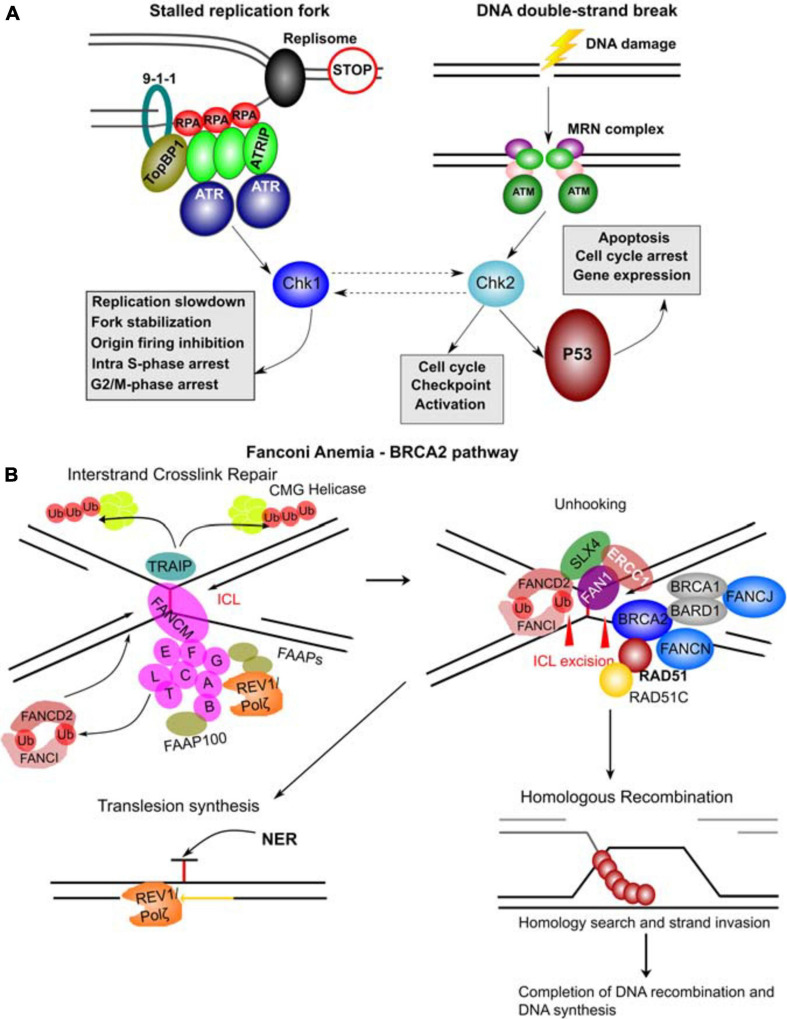
Molecular complexes formed and DNA repair pathways mediated by CPG proteins. **(A)** Mechanisms of ATR and ATM activation by different types of DNA damage. Stalled replication forks resulting from a blockage of replisome progression (indicated by the “STOP” diagram). The single-stranded DNA (ssDNA) region is recognized by Replication Protein A (RPA) forming the filaments which can then recruit ATR-ATRIP (ATR Interacting Protein) complexes. The RAD17-RFC complex (not shown) loads the 9-1-1 complex (RAD9-RAD1-HUS1) onto the ssDNA-dsDNA junction. RAD9 S387 phosphorylation provides a binding site for DNA topoisomerase 2-binding protein 1 (TopBP1), which in turn binds and activates ATR kinase. ATR phosphorylates and activates Chk1 kinase mediating many of its functional effects and directly phosphorylates many other target proteins. DNA double-strand breaks results in a complex signaling cascade resulting in binding of the MRN complex (MRE11-RAD50-NBN) to the DNA ends. MRN provides a DNA damage sensor platform for recruitment and activation of ATM, which then phosphorylates multiple targets including Chk2 responsible for cell cycle checkpoint activation and p53 activation. Chk2 and Chk1 can also functionally interact (hashed lines with arrows). p53 activation leads to apoptosis, cell cycle arrest and gene expression changes. **(B)** FA – BRCA2 pathway. A major role of this pathway is to respond to inter-strand crosslinks (ICL), which can be especially problematic during DNA replication since ICLs prevent replication fork convergence. At such blocked replication forks, TRAIP ubiquitin ligase ubiquitinates the CMG (CDC45-MCM-GINS) Helicase leading to its unloading. The ICL itself is recognized by FANCM / FAAP24 complex. FANCM then recruits the core FA complex components and FAAPs (FA associated proteins). E2 ubiquitin-conjugating enzyme FANCT and E3 ubiquitin ligase FANCL then cooperate to perform mono-ubiquitination of FANCD2 and FANCI forming the ID2 complex. Monoubiquitinated ID2 complex gets recruited the ICL lesion. In the process of unhooking, DNA near the ICL gets processed by nucleases to enable specific DNA repair pathways. The strand with the ICL structure gets repaired by Nucleotide Excision Repair (NER) and the translesion synthesis by REV1/Polζ, which gets recruited by the FA core complex. ID2 recruits FANCP (SLX4), FANCQ (ERCC1) and the nuclease FAN1 to mediate the ICL incision. The ID2 also interacts with BRCA2 (FANCD1), which gets recruited to the parts of blocked replication forks that will have to go through DNA recombination. BRCA2 in complex with BRCA1, BARD1, FANCN, FANCJ, RAD51 and RAD51C as well as other factors leads to loading of RAD51 into resected ssDNA regions, which can then participate in homologous recombination (HR). The figure was constructed based on the information in several recent reviews (Awasthi et al., 2016; Fradet-Turcotte et al., 2016; Bonilla et al., 2020; Liu et al., 2020).

ATM (A-T mutated) and ATR (A-T and Rad3 mutated) are key regulators of the initiation of the DDR (Awasthi et al., 2016). *ATM* mutations are responsible for Ataxia-Telangectasia (A-T) which is characterized by progressive ataxia, dilatation of small vessels (telangiectasia), immune defects, genome instability and malignancy while *ATR* mutations are implicated in Seckel syndrome which is associated with dwarfism, microcephaly, growth defects and intellectual disabilities (Weber and Ryan, 2015). ATM is activated by DNA DSBs while ATR is activated primarily by single-stranded DNA filaments resulting from replication stress (Figure 7A). Both ATM and ATR belong to the PI3K-related kinase (PIKK) family and upon activation they phosphorylate many target proteins including p53, Chk2 and Chk1 kinases, which mediate many of the downstream effects of ATM and ATR (Awasthi et al., 2016; Figure 7). *CHEK2* is also an established CPG confirming the importance of this pathway in cancer.

Zebrafish ATM is highly conserved in specific domains, exhibits a broad pattern of expression at early stages and morpholino knockdown results in increased sensitivity to γ-irradiation (Imamura and Kishi, 2005). Further, in *prim1* (DNA primase subunit 1) zebrafish mutants, knockdown of *atm* or *atr* is too toxic for retinal development, but retinal apoptosis due to DNA damage from faulty DNA replication is mostly suppressed with chemical inhibition of ATM (via KU55933) or ATM/ATR (via CGK733), or knockdown of *chk2* (Yamaguchi et al., 2008). Similar observations were made in the *ugly duckling* (*udu*) zebrafish mutant now mapped to the *gon4l* gene which has been recently implicated in regulating several pleotropic mechanisms including DNA repair and genome stability (Lim et al., 2009; Tsai et al., 2020). p53-mediated apoptosis in this mutant could be successfully inhibited by either knockdown of *tp53*, *chk2* or chemical inhibition of ATM.

Even though zebrafish ATR does not appear to control the DDR directly, it has been linked to cilia formation in Kuppfer’s vesicle cells involved in establishment of left-right asymmetry (Stiff et al., 2016). ATR knockdown in zebrafish reduces the size of the Kuppfer’s vesicle, shortens cilia in this area, reduces *gli1* levels, a Hedgehog pathway target, and induces p53 targets *p21* and *puma*. The authors linked p53 target induction to Hedgehog signaling inhibition, but it is more likely to be a direct ATR knockdown effect due to limited evidence of Hedgehog pathway inhibition. Morphologically, ATR knockdown produced left-right asymmetry defects, curved bodies, small eyes and narrow heads in zebrafish embryos, phenotypes supportive of significant Hedgehog signaling inactivation. As of this review, there are no characterized zebrafish mutants of either *atm* or *atr*. Based on the above studies, null mutants may have strong phenotypes and generation of both null and precise point mutants would help identify the spectrum of potential developmental and cancer predisposition phenotypes of *ATM* and *ATR* mutations in zebrafish to discern the relative roles of these kinases in genome stability maintenance and tumor suppression.

#### Fanconi Anemia Genes

Fanconi anemia is a genetic disorder caused by mutations in a group of genes involved in several aspects of DNA repair. Collectively known as the Fanconi anemia pathway, this group (22 known members) of FA proteins forms multiple complexes and mediate DNA interstrand crosslink (ICL) repair resulting from endogenous aldehydes or exogenous crosslinking agents (Liu et al., 2020). Briefly, this involves fork convergence, ICL recognition by the FA core complex, and removal of DNA replication components, involving FA genes such as *BRCA2*, *RAD51* and others (Figure 7B). FA is an IBMFS with disease characteristics that include cancer predisposition, fertility issues, growth retardation, and congenital abnormalities affecting many tissue types (Ramanagoudr-Bhojappa et al., 2018). Individuals with FA have defective HSCs that result in aplastic anemia due to BMF, MDS, and AML (Kutler et al., 2003). The frequency of BMF is estimated at 80% in affected individuals, of whom 30% develop hematologic and solid tumors including leukemias, head and neck carcinomas, liver tumors and gynecologic malignancies by 40 years of age with rates several hundred-fold higher than the general population and the age of onset frequently in childhood or early adulthood (Kutler et al., 2003; Liu et al., 2020).

Most FA genes are highly conserved in zebrafish (Titus et al., 2009) and the most comprehensive study of 17 FA genes (*fanca*, *fancb*, *fancc*, *fancd1/brca2*, *fancd2*, *fance, fancf, fancg, fanci, fancj/brip1, fancl, fancm, fancn/palb2, fanco/rad51c, fancp/slx4*, *fancq/ercc4*, *fanct/ube2t* and 2 FA-associated genes: *faap100* and *faap24)* revealed that FA mutants do not exhibit overt morphological phenotypes during embryonic development (Ramanagoudr-Bhojappa et al., 2018). However, adult FA zebrafish experience a female-to-male sex reversal as mutants in 12 genes had a complete reversal while 5 others had a partial reversal. This reversal has complicated certain phenotypic analyses due to the need for crosses with heterozygous females, which prevents generation of maternal-zygotic mutants. Additionally, 11 out of 17 mutants have some degree of hypersensitivity to treatment with diepoxybutane (DEB), which serves as a diagnostic test for FA gene mutations. *fancp* mutants exhibit a decreased body size but no FA mutants demonstrate blood-related phenotypes or increased cancer susceptibility (Ramanagoudr-Bhojappa et al., 2018). Previous work in *fancl* zebrafish mutants similarly demonstrated female-to-male sex reversal, which mechanistically was connected to germ cell apoptosis mediated by p53 activation (Rodríguez-Marí et al., 2010). This reversal was blocked in the *fancl^–/–^; tp53^–/–^* mutants, confirming the role of p53 in this process and suggested a model where lack of oocyte survival generates signals for testis development and overall male sex determination in *fancl^–/–^* mutants.

Fanconi anemia genes such as *BRCA2/FANCD1, RAD51C* and *RAD51*, homologous to the bacterial RecA, are involved in repair of DNA DSBs by homologous recombination (Bonilla et al., 2020). BRCA2 is also more broadly important for genome stability due to its involvement in DNA replication, cell cycle progression and telomere homeostasis (Fradet-Turcotte et al., 2016). Zebrafish *brca2/fancd1* has received a significant amount of research attention which has revealed its important functions in genome stability, germ cell development, kidney development and tumor suppression. Female-to-male reversal and male infertility is observed in homozygous *brca2* zebrafish mutants (Q658X and ZM_00057434 referred to as *brca2^–/–^* and both have a disruption in exon 11 of *brca2* which is commonly observed in hereditary breast and ovarian tumors) due to the disruptions in germ cell meiosis after being correctly specified in development (Shive et al., 2010; Rodríguez-Marí et al., 2011). Loss of *tp53* rescues ovary development but not male fertility. Apoptosis is dramatically increased after DEB treatment in *brca2^–/–^* zebrafish embryos and *brca2^–/–^* mutant cell lines have higher rates of chromosomal aberrations, apoptosis and slower growth compared to wild-type (Rodríguez-Marí et al., 2011). These *brca2* mutant studies represent the first FA zebrafish cancer susceptibility models as both the infertile *brca2^–/–^* males developed testicular cancer, and *tp53* and *brca2* double heterozygotes (*tp53^+/^*^*M214K*^; *brca2^+/–^*) or homozygotes (*tp53*^*M214K/M214K*^; *brca2^–/–^*) had accelerated cancer development by about 5 months (Rodríguez-Marí et al., 2011). To further analyze tumor development, *brca2*^*Q658X*^ mutants were crossed to a *tp53* mutant background to drive tumor formation (Shive et al., 2014). After a > 2-year follow-up, all lines, *brca2*^+/+^; *tp53^+/^*^*M214K*^, *brca2*^+/Q658X^; *tp53^+/^*^*M214K*^ and *brca2*^*Q658X/Q658X*^; *tp53^+/^*^*M214K*^, had a tumor incidence of 80–100% and *tp53* LOH was observed nearly universally in the *brca2*^+/+^; *tp53^+/^*^*M214K*^ tumors while it was only observed in 30% of the *brca2*^*Q658X/Q658X*^; *tp53^+/^*^*M214K*^ tumors, suggesting that *brca2* homozygous mutants undergo other genetic changes that drive tumorigenesis (Shive et al., 2014). Additionally, the creation of the zebrafish *zeppelin* (*zep*) mutant, mapped to *brca2* by whole-genome sequencing, has revealed that *brca2* also plays a role in kidney development (Kroeger et al., 2017).

*RAD51/FANCR* has recently been associated with FA and provides genetic support to well-known interactions between RAD51 and BRCA2 (Bonilla et al., 2020). This functional interaction is well-conserved in zebrafish as antibody staining in embryos show that endogenous rad51 forms foci upon induction of DNA DSBs, but these foci do not form in *brca2^–/–^* mutants and are reduced in *brca2^+/–^* embryos (Vierstraete et al., 2017). A recent zebrafish study showed that *rad51* null embryos exhibit both increased chromosomal abnormalities after DEB treatment and increased sensitivity to gamma irradiation, consistent with the designation of *RAD51* as an FA pathway gene (Botthof et al., 2017). Zebrafish *rad51l1* mutants, a *rad51* paralog, are synthetically lethal when combined with *rad51* mutant, demonstrating the redundancy of their functions. In contrast to all other FA gene mutants, *rad51^–/–^* mutants exhibit decreased staining for HSC markers during larval stages and lower total cell numbers in kidneys in adults, both of which are rescued by *tp53* loss (Botthof et al., 2017).

#### DNA Mismatch Repair

The DNA mismatch repair (MMR) pathway maintains chromosome stability and prevents increases in the rate of mutations, therefore it is of great significance for the maintenance of genome stability (Ijsselsteijn et al., 2020). Indeed, genes involved in the MMR pathway, including *MLH1, MSH2, MSH6, PMS1, PMS2*, are known CPGs and their mutations are responsible for Lynch syndrome (hereditary non-polyposis colorectal cancer) (Rahman, 2014). The zebrafish *mlh1* mutant shows the importance of mlh1 for chromosome segregation in meiosis during gametogenesis as mutant males are infertile and exhibit abnormal testis histology, accumulation of spermatocytes and apoptosis (Feitsma et al., 2007) with a < 1% fertilization rate, as well as higher mutational burden in the resulting embryos (Leal et al., 2008). *mlh1* mutant females are fertile when bred with wild-type males, but still frequently produced aneuploid and triploid embryos. These phenotypes are fully consistent with mouse models of MMR defects and possible links of MMR gene defects to human male infertility (Mukherjee et al., 2010). Further characterization of *mlh1*, *msh2* and *msh6* mutants shows microsatellite instability in the progeny from mutant males and revealed increased cancer incidence in mutant adults with tumors mainly characterized as neurofibromas and MPNSTs (Feitsma et al., 2008).

#### CPGs of Diverse Functions Contribute to Genome Stability

The genes discussed so far in this section are directly involved in DNA repair pathways. However, given the complexity of this process and its regulatory mechanisms, it can be expected that additional genes will be implicated in tumor suppression that have indirect or unexpected roles in genome stability. We will discuss several case studies highlighting how the zebrafish model has been employed to examine such genes.

ATRX is a SWI/SNF-like chromatin remodeler specialized in depositing H3.3 histone onto genomic DNA in a complex with the histone chaperone DAXX. Loss of ATRX is associated with both alternative lengthening of telomeres, thus promoting cell immortalization, and a wide range of cancer types, especially those of the nervous system, such as gliomas and neuroendocrine tumors (Nye et al., 2018). However, an recently-identified interaction between ATRX and FANCD2 implicates ATRX and DAXX in repair mechanisms of DNA DSBs, suggesting a direct and active role of ATRX in the DDR (Raghunandan et al., 2020). Although *atrx* null mutant (*atrx^–/–^*) zebrafish do not survive past larval stages, blood development in mutant embryos is relatively normal with the exception of a single globin gene and more spherical erythrocytes (Oppel et al., 2019). Lethality of *atrx* loss required the authors to investigate its cancer-promoting properties in highly sensitized tumor-prone mutants, *tp53^–/–^; nf1b^–/–^; nf1a^+/–^; atrx^+/–^* and *tp53^–/–^; nf1b^–/–^; nf1a^+/+^; atrx^+/–^*. Heterozygous *atrx* loss did not increase the rate of tumor formation but led to a wider spectrum of tumor types, *tert* down-regulation, lengthening of telomeres and up-regulation of polycomb repressive complex 2 genes compared to *atrx^+/+^* tumors. This model represents an important step for *atrx* investigation in zebrafish and may be further improved by tissue-specific or conditional approaches to *atrx* inactivation to circumvent larval lethality of this mutant.

The designation of *DICER1* and *DROSHA* as CPGs is not surprising given their fundamental roles in microRNA (miRNA) biogenesis as miRNA genes are well-known oncogenes and tumor suppressors (Hammond, 2015) and there is a diverse spectrum of cancers observed in DICER1 syndrome (Robertson et al., 2018). However, over the last decade, small RNAs and lncRNAs have been implicated in the DDR (Vågbø and Slupphaug, 2020). Genomic DDR foci produce DICER1- and DROSHA-dependent small RNAs (20–35 nucleotides), which are required for full DDR activation in mammalian cells and zebrafish (Francia et al., 2012). Further, *dicer1* knockdown in zebrafish strongly disrupts the DDR based on reductions in ATM and γ-H2AX phosphorylation in a cell-autonomous manner based on the aberrant DDR in transplanted *dicer1* morphant cells (Francia et al., 2012). This suggests that DICER1 and DROSHA are part of the DNA repair pathway and further evaluation is needed from the perspectives of both miRNA biogenesis and genome stability maintenance.

The von Hippel-Lindau protein (pVHL) mainly functions in the cellular response to hypoxia where it is responsible for poly-ubiquitination of Hypoxia Inducible Factor (HIF) proteins 1 and 2. Under normoxic conditions, HIF1/2 are hydroxylated and degraded; however, they become less hydroxylated under hypoxic conditions and accumulate, causing transactivation of their target genes. Loss of pVHL leads to a constitutively active hypoxic response and is the cause of von Hippel Lindau disease. Affected individuals develop retinal and central nervous system vascular tumors known as hemangioblastomas, pancreatic and kidneys cysts, pheochromocytoma, and clear cell renal cell carcinomas (ccRCC) (Gossage et al., 2015). Previous work in ccRCC cell lines demonstrates the importance of pVHL for DNA DSB repair (Metcalf et al., 2014). *VHL* has two paralogs in zebrafish, *vhl* and *vll*, and zebrafish pVHL mutants demonstrate that *vhl* gene loss is mainly responsible for HIF signaling activation, which is significantly enhanced upon *vll* loss, while *vll* is important for genome stability maintenance (Kim et al., 2020). The *vhl^–/–^*; *vll^–/–^* double mutants have reduced p53 activation and are resistance to apoptosis following the induction of genotoxic stimuli which was phenocopied by Hif activation. Additionally, genotoxic-induced apoptosis in *brca2^–/–^* mutants is rescued by *vhl* loss. Thus, these results firmly establish *vhl* and *vll* as VHL homologs with divided but complementary functions that can enable the creation of cancer models involving these genes (Kim et al., 2020).

## Conclusion

Zebrafish models are amenable to a variety of genetic technologies and represent a powerful *in vivo* tool to investigate the roles and mechanisms of CPGs in development and tumorigenesis. We have reviewed how the zebrafish is well-positioned for preclinical studies focused on identifying novel mutations and genes, as well as elucidating how their molecular mechanisms contribute to biological functioning and cancer development. One major advantage of the zebrafish model is the more rapid and less expensive generation of genetic disease models than is possible in the mouse. Zebrafish also provide excellent opportunities for *in vivo* imaging throughout development and to some extent in adults. Additionally, mutations of certain genes are often more tolerable in zebrafish than in the mouse which is partially explained by the partial genome duplication that occurred in zebrafish and as such, zebrafish models often survive longer and allow for more in-depth studies. This phenomenon also provides the ability to investigate the effect of multiple modifier genes through the creation of compound mutants, especially for CPGs. One common CPG/modifier often investigated is *tp53* due to the prominent role of *tp53* in tumor suppression. Several of the cancer predisposition models we have highlighted investigated the role of p53 in disease progression and have employed *tp53* mutants to facilitate tumor development in their models.

In this way, zebrafish models have tremendous potential to provide preclinical insights to change the natural history of life-threatening cancer predisposition disorders. Mutations in CPGs predispose individuals to an increased risk of cancer via their key roles in both development and homeostatic control/maintenance, especially during times of cellular stress like DNA damage or energy depletion. Thus, the generation and characterization of zebrafish models has tremendous utility for identifying novel therapies for these rare but devastating syndromes. Further, knowledge gained from zebrafish models also holds great significance to the broader cancer research community, as biological insights about CPG mechanisms will also reveal the biological processes underlying sporadic tumorigenesis. However, one current limitation is that several of the aforementioned zebrafish disease models are knockouts while their corresponding syndrome is more typically caused by a missense mutation that does not result in protein truncation; thus, these models may not fully represent the genetic underpinnings of the disease nor the resulting protein interactions. This was likely due to the availability/usability of genome editing technologies at the time of model creation, but the recent optimization of several genome editing techniques, as well as soon anticipated-in-zebrafish technologies including prime editing, will aid future researchers to more easily and efficiently develop more representative disease-specific genetic models. Additionally, once established, genetic zebrafish models can serve as a drug screening platform to rapidly and efficiently identify novel therapies. However, to date, zebrafish cancer predisposition models have been employed predominantly in proof-of-principle drug studies with known molecular targets to highlight genotype-phenotype correlations. Thus, this represents an exciting and powerful next step for many of these models to provide clinical insights and reveal new avenues of therapeutic intervention.

## Author Contributions

KK, KC, and SP wrote and edited the manuscript and generated the figures and tables. JB edited the manuscript. All authors contributed to the article and approved the submitted version.

## Conflict of Interest

The authors declare that the research was conducted in the absence of any commercial or financial relationships that could be construed as a potential conflict of interest.

## References

[B1] Al-Qahtani F. S. (2010). Congenital amegakaryocytic thrombocytopenia: a brief review of the literature. *Clin. Med. Insights Pathol.* 3 25–30. 10.4137/CPath.S4972 21151552 PMC2999995

[B2] Alter B. P. Giri N. Savage S. A. Rosenberg P. S. (2009). Cancer in dyskeratosis congenita. *Blood* 113 6549–6557. 10.1182/blood-2008-12-192880 19282459 PMC2710915

[B3] Anchelin M. Alcaraz-Pérez F. Martínez C. M. Bernabé-García M. Mulero V. Cayuela M. L. (2013). Premature aging in telomerase-deficient zebrafish. *Dis. Model. Mech.* 6 1101–1112. 10.1242/dmm.011635 23744274 PMC3759330

[B4] Antony-Debré I. Duployez N. Bucci M. Geffroy S. Micol J. B. Renneville A. (2016). Somatic mutations associated with leukemic progression of familial platelet disorder with predisposition to acute myeloid leukemia. *Leukemia* 30 999–1002. 10.1038/leu.2015.236 26316320

[B5] Anzalone A. V. Randolph P. B. Davis J. R. Sousa A. A. Koblan L. W. Levy J. M. (2019). Search-and-replace genome editing without double-strand breaks or donor DNA. *Nature* 576 149–157. 10.1038/s41586-019-1711-4 31634902 PMC6907074

[B6] Aoki K. Tamai Y. Horiike S. Oshima M. Taketo M. M. (2003). Colonic polyposis caused by mTOR-mediated chromosomal instability in Apc+/Δ716 Cdx2+/- compound mutant mice. *Nat. Genet.* 35 323–330. 10.1038/ng1265 14625550

[B7] Aoki Y. Niihori T. Kawame H. Kurosawa K. Ohashi H. Tanaka Y. (2005). Germline mutations in HRAS proto-oncogene cause Costello syndrome. *Nat. Genet.* 37 1038–1040. 10.1038/ng1641 16170316

[B8] Avagyan S. Mannherz W. Zon L. I. (2017). Visualizing clonal hematopoiesis associated with gata2 deficiency in zebrafish using color-barcoding. *Blood* 130:4237. 10.1182/blood.V130.Suppl_1.4237.4237

[B9] Awasthi P. Foiani M. Kumar A. (2016). ATM and ATR signaling at a glance. *J. Cell Sci.* 129:1285. 10.1242/jcs.188631 26979625

[B10] Bai H. Liu L. An K. Lu X. Harrison M. Zhao Y. (2020). CRISPR/Cas9-mediated precise genome modification by a long ssDNA template in zebrafish. *BMC Genomics* 21:67. 10.1186/s12864-020-6493-4 31964350 PMC6974980

[B11] Berghmans S. Murphey R. D. Wienholds E. Neuberg D. Kutok J. L. Fletcher C. D. M. (2005). Tp53 mutant zebrafish develop malignant peripheral nerve sheath tumors. *Proc. Natl. Acad. Sci. U.S.A.* 102 407–412. 15630097 10.1073/pnas.0406252102 PMC544293

[B12] Bigley V. Collin M. (2011). Dendritic cell, monocyte, B and NK lymphoid deficiency defines the lost lineages of a new GATA-2 dependent myelodysplastic syndrome. *Haematologica* 96 1081–1083. 10.3324/haematol.2011.048355 21810969 PMC3148897

[B13] Bond G. L. Hu W. Bond E. E. Robins H. Lutzker S. G. Arva N. C. (2004). A single nucleotide polymorphism in the MDM2 promoter attenuates the p53 tumor suppressor pathway and accelerates tumor formation in humans. *Cell* 119 591–602. 10.1016/j.cell.2004.11.022 15550242

[B14] Bonetti M. Overman J. P. Tessadori F. Noël E. Bakkers J. den Hertog J. (2014a). Noonan and LEOPARD syndrome Shp2 variants induce heart displacement defects in zebrafish. *Development* 141 1961–1970. 10.1242/dev.106310 24718990

[B15] Bonetti M. Rodriguez-Martinez V. Overman J. P. Overvoorde J. Van Eekelen M. Jopling C. (2014b). Distinct and overlapping functions of ptpn11 genes in zebrafish development. *PLoS One* 9:e94884. 10.1371/journal.pone.0094884 24736444 PMC3988099

[B16] Bonilla B. Hengel S. Grundy M. Bernstein K. (2020). RAD51 gene family structure and function. *Annu. Rev. Genet.* 54 1–22. 10.1146/annurev-genet-021920-092410 32663049 PMC7703940

[B17] Boocock G. R. B. Morrison J. A. Popovic M. Richards N. Ellis L. Durie P. R. (2003). Mutations in SBDS are associated with shwachman–diamond syndrome. *Nat. Genet.* 33 97–101. 10.1038/ng1062 12496757

[B18] Botthof J. G. Bielczyk-Maczynska E. Ferreira L. Cvejic A. (2017). Loss of the homologous recombination gene rad51 leads to Fanconi anemia-like symptoms in zebrafish. *Proc. Natl. Acad. Sci. U. S. A.* 114 E4452–E4461. 10.1073/pnas.1620631114 28512217 PMC5465903

[B19] Brannan C. I. Perkins A. S. Vogel K. S. Ratner N. Nordlund M. L. Reid S. W. (1994). Targeted disruption of the neurofibromatosis type-1 gene leads to developmental abnormalities in heart and various neural crest-derived tissues. *Genes Dev.* 8 1019–1029. 10.1101/gad.8.9.1019 7926784

[B20] Burroughs L. Woolfrey A. Shimamura A. (2009). Shwachman-diamond syndrome: a review of the clinical presentation, molecular pathogenesis, diagnosis, and treatment. *Hematol. Oncol. Clin. North Am.* 23 233–248. 10.1016/j.hoc.2009.01.007 19327581 PMC2754297

[B21] Butko E. Distel M. Pouget C. Weijts B. Kobayashi I. Ng K. (2015). Gata2b is a restricted early regulator of hemogenic endothelium in the zebrafish embryo. *Development* 142 1050–1061. 10.1242/dev.119180 25758220 PMC4360177

[B22] Cadwell C. Zambetti G. P. (2001). The effects of wild-type p53 tumor suppressor activity and mutant p53 gain-of-function on cell growth. *Gene* 277 15–30. 10.1016/S0378-1119(01)00696-5 11602342

[B23] Carneiro M. C. De Castro I. P. Ferreira M. G. (2016). Telomeres in aging and disease: lessons from zebrafish. *Dis. Model. Mech.* 9 737–748. 10.1242/dmm.025130 27482813 PMC4958310

[B24] Chen J. S. Dagdas Y. S. Kleinstiver B. P. Welch M. M. Sousa A. A. Harrington L. B. (2017). Enhanced proofreading governs CRISPR–Cas9 targeting accuracy. *Nature* 550 407–410. 10.1038/nature24268 28931002 PMC5918688

[B25] Chi Y. Huang Z. Chen Q. Xiong X. Chen K. Xu J. (2018). Loss of runx1 function results in B cell immunodeficiency but not T cell in adult zebrafish. *Open Biol.* 8:180043. 10.1098/rsob.180043 30045885 PMC6070721

[B26] Choi M. Y. Cho M.-H. Chang H. J. Lee S.-N. Lee K. E. (2019). Investigation of enhanced antitumor effects via co-inhibition of Wnt/β-catenin and PI3K/Akt/mTOR signaling pathways in human gastric cancer. *J. Clin. Oncol.* 37 (15_suppl):e15553. 10.1200/JCO.2019.37.15_suppl.e15553

[B27] Choorapoikayil S. Kuiper R. V. de Bruin A. den Hertog J. (2012). Haploinsufficiency of the genes encoding the tumor suppressor Pten predisposes zebrafish to hemangiosarcoma. *Dis. Model. Mech.* 5 241–247. 10.1242/dmm.008326 22071262 PMC3291645

[B28] Choorapoikayil S. Weijts B. Kers R. de Bruin A. den Hertog J. (2013). Loss of Pten promotes angiogenesis and enhanced vegfaa expression in zebrafish. *Dis. Model. Mech.* 6 1159–1166. 10.1242/dmm.012377 23720233 PMC3759335

[B29] Cichowski K. Jacks T. (2001). NF1 tumor suppressor gene function. *Cell* 104 593–604. 10.1016/S0092-8674(01)00245-8 11239415

[B30] Cole H. N. Rauschkolb J. E. Toomey J. (1930). Dyskeratosis congenita with pigmentation, dystrophia unguis and leukokeratosis oris. *Arch. Derm. Syphilol.* 21 71–95. 10.1001/archderm.1930.01440070079008 14360753

[B31] Crino P. B. Nathanson K. L. Henske E. P. (2006). The tuberous sclerosis complex. *N. Engl. J. Med.* 355 1345–1356. 10.1056/NEJMra055323 17005952

[B32] Cvejic A. Hall C. Bak-Maier M. Flores M. V. Crosier P. Redd M. J. (2008). Analysis of WASp function during the wound inflammatory response - live-imaging studies in zebrafish larvae. *J. Cell Sci.* 121 3196–3206. 10.1242/jcs.032235 18782862

[B33] Dai Y. Zhu L. Huang Z. Zhou M. Jin W. Liu W. (2016). Cebpα is essential for the embryonic myeloid progenitor and neutrophil maintenance in zebrafish. *J. Genet. Genomics* 43 593–600. 10.1016/j.jgg.2016.09.001 27751705

[B34] d’Amora M. Giordani S. (2018). The utility of zebrafish as a model for screening developmental neurotoxicity. *Front. Neurosci.* 12:976. 10.3389/fnins.2018.00976 30618594 PMC6305331

[B35] Dasgupta B. Dugan L. L. Gutmann D. H. (2003). The neurofibromatosis 1 gene product neurofibromin regulates pituitary adenylate cyclase-activating polypeptide-mediated signaling in astrocytes. *J. Neurosci.* 23 8949–8954. 10.1523/JNEUROSCI.23-26-08949.2003 14523097 PMC6740397

[B36] Davidson A. J. Zon L. I. (2004). The ‘definitive’ (and ‘primitive’) guide to zebrafish hematopoiesis. *Oncogene* 23 7233–7246. 10.1038/sj.onc.1207943 15378083

[B37] Delacruz R. G. C. Sandoval I. T. Chang K. Miller B. N. Reyes-Uribe L. Borras E. (2019). Functional characterization of CNOT3 variants identified in familial adenomatous polyposis adenomas. *Oncotarget* 10 3939–3951. 10.18632/oncotarget.27003 31231471 PMC6570471

[B38] Deshaies R. J. Joazeiro C. A. P. (2009). RING domain E3 ubiquitin ligases. *Annu. Rev. Biochem.* 78 399–434. 10.1146/annurev.biochem.78.101807.093809 19489725

[B39] Di Micco R. Fumagalli M. Cicalese A. Piccinin S. Gasparini P. Luise C. (2006). Oncogene-induced senescence is a DNA damage response triggered by DNA hyper-replication. *Nature* 444 638–642. 10.1038/nature05327 17136094

[B40] DiBella L. M. Park A. Sun Z. (2009). Zebrafish Tsc1 reveals functional interactions between the cilium and the TOR pathway. *Hum. Mol. Genet.* 18 595–606. 10.1093/hmg/ddn384 19008302 PMC2722215

[B41] Dickinson R. E. Griffin H. Bigley V. Reynard L. N. Hussain R. Haniffa M. (2011). Exome sequencing identifies GATA-2 mutation as the cause of dendritic cell, monocyte, B and NK lymphoid deficiency. *Blood* 118 2656–2658. 10.1182/blood-2011-06-360313 21765025 PMC5137783

[B42] Dobrzycki T. Mahony C. B. Krecsmarik M. Koyunlar C. Rispoli R. Peulen-Zink J. (2020). Deletion of a conserved Gata2 enhancer impairs haemogenic endothelium programming and adult zebrafish haematopoiesis. *Commun. Biol.* 3:71. 10.1038/s42003-020-0798-3 32054973 PMC7018942

[B43] Duran A. Amanchy R. Linares J. F. Joshi J. Abu-Baker S. Porollo A. (2011). p62 is a key regulator of nutrient sensing in the mTORC1 pathway. *Mol. Cell* 44 134–146. 10.1016/j.molcel.2011.06.038 21981924 PMC3190169

[B44] Eisinger A. L. Nadauld L. D. Shelton D. N. Peterson P. W. Phelps R. A. Chidester S. (2006). The adenomatous polyposis coli tumor suppressor gene regulates expression of cyclooxygenase-2 by a mechanism that involves retinoic acid. *J. Biol. Chem.* 281 20474–20482. 10.1074/jbc.M602859200 16699180

[B45] El-Brolosy M. A. Kontarakis Z. Rossi A. Kuenne C. Günther S. Fukuda N. (2019). Genetic compensation triggered by mutant mRNA degradation. *Nature* 568 193–197. 10.1038/s41586-019-1064-z 30944477 PMC6707827

[B46] Eng C. (2003). PTEN: one gene, many syndromes. *Hum. Mutat.* 22 183–198. 10.1002/humu.10257 12938083

[B47] Evangelisti C. Chiarini F. Cappellini A. Paganelli F. Fini M. Santi S. (2020). Targeting Wnt/β-catenin and PI3K/Akt/mTOR pathways in T-cell acute lymphoblastic leukemia. *J. Cell. Physiol.* 235 5413–5428. 10.1002/jcp.29429 31904116

[B48] Fahsold R. Hoffmeyer S. Mischung C. Gille C. Ehlers C. Kücükceylan N. (2000). Minor lesion mutational spectrum of the entire NF1 gene does not explain its high mutability but points to a functional domain upstream of the GAP-related domain. *Am. J. Hum. Genet.* 66 790–818. 10.1086/302809 10712197 PMC1288164

[B49] Faucherre A. Taylor G. S. Overvoorde J. Dixon J. E. den Hertog J. (2008). Zebrafish pten genes have overlapping and non-redundant functions in tumorigenesis and embryonic development. *Oncogene* 27 1079–1086. 10.1038/sj.onc.1210730 17704803

[B50] Feitsma H. Kuiper R. V. Korving J. Nijman I. J. Cuppen E. (2008). Zebrafish with mutations in mismatch repair genes develop neurofibromas and other tumors. *Cancer Res.* 68 5059–5066. 10.1158/0008-5472.CAN-08-0019 18593904

[B51] Feitsma H. Leal M. C. Moens P. B. Cuppen E. Schulz R. W. (2007). Mlh1 deficiency in zebrafish results in male sterility and aneuploid as well as triploid progeny in females. *Genetics* 175 1561–1569. 10.1534/genetics.106.068171 17237513 PMC1855116

[B52] Fenton H. Carlile B. Montgomery E. A. Carraway H. Herman J. Sahin F. (2006). LKB1 protein expression in human breast cancer. *Appl. Immunohistochem. Mol. Morphol.* 14 146–153. 10.1097/01.pai.0000176157.07908.20 16785781

[B53] Fodde R. Edelmann W. Yang K. van Leeuwen C. Carlson C. Renault B. (1994). A targeted chain-termination mutation in the mouse Apc gene results in multiple intestinal tumors. *Proc. Natl. Acad. Sci. U.S.A.* 91 8969–8973. 10.1073/pnas.91.19.8969 8090754 PMC44728

[B54] Fodde R. Smits R. Clevers H. (2001). APC, Signal transduction and genetic instability in colorectal cancer. *Nat. Rev. Cancer* 1 55–67. 10.1038/35094067 11900252

[B55] Fradet-Turcotte A. Sitz J. Grapton D. Orthwein A. (2016). BRCA2 functions: from DNA repair to replication fork stabilization. *Endocr. Relat. Cancer* 23 T1–T17. 10.1530/ERC-16-0297 27530658

[B56] Francia S. Michelini F. Saxena A. Tang D. De Hoon M. Anelli V. (2012). Site-specific DICER and DROSHA RNA products control the DNA-damage response. *Nature* 488 231–235. 10.1038/nature11179 22722852 PMC3442236

[B57] Fuchs O. Provaznikova D. Kocova M. Kostecka A. Cvekova P. Neuwirtova R. (2008). CEBPA polymorphisms and mutations in patients with acute myeloid leukemia, myelodysplastic syndrome, multiple myeloma and non-Hodgkin’s lymphoma. *Blood Cells Mol. Dis.* 40 401–405. 10.1016/j.bcmd.2007.11.005 18182175

[B58] Gao J. Aksoy B. A. Dogrusoz U. Dresdner G. Gross B. Sumer S. O. (2013). Integrative analysis of complex cancer genomics and clinical profiles using the cBioPortal. *Sci. Signal.* 6:l1. 10.1126/scisignal.2004088 23550210 PMC4160307

[B59] Gao X. Johnson K. D. Chang Y. I. Boyer M. E. Dewey C. N. Zhang J. (2013). Gata2 cis-element is required for hematopoietic stem cell generation in the mammalian embryo. *J. Exp. Med.* 210 2833–2842. 10.1084/jem.20130733 24297994 PMC3865483

[B60] Gao X. Huang S.-S. Qiu S.-W. Su Y. Wang W.-Q. Xu H.-Y. (2020). Congenital sensorineural hearing loss as the initial presentation of PTPN11 -associated Noonan syndrome with multiple lentigines or Noonan syndrome: clinical features and underlying mechanisms. *J. Med. Genet.* 10.1136/jmedgenet-2020-106892 Online ahead of print 32737134

[B61] Garber J. E. Offit K. (2005). Hereditary cancer predisposition syndromes. *J. Clin. Oncol.* 23 276–292. 10.1200/JCO.2005.10.042 15637391

[B62] Germeshausen M. Ballmaier M. Welte K. (2006). MPL mutations in 23 patients suffering from congenital amegakaryocytic thrombocytopenia: the type of mutation predicts the course of the disease. *Hum. Mutat.* 27:296. 10.1002/humu.9415 16470591

[B63] Giardiello F. M. Welsh S. B. Hamilton S. R. Offerhaus G. J. A. Gittelsohn A. M. Booker S. V. (1987). Increased risk of cancer in the peutz–jeghers syndrome. *N. Engl. J. Med.* 316 1511–1514. 10.1056/NEJM198706113162404 3587280

[B64] Gillis W. Q. St John J. Bowerman B. Schneider S. Q. (2009). Whole genome duplications and expansion of the vertebrate GATA transcription factor gene family. *BMC Evol. Biol.* 9:207. 10.1186/1471-2148-9-207 19695090 PMC2857956

[B65] Gioacchino E. Koyunlar C. de looper H. Peulen J. Bosch D. Hoogenboezem R. (2019). Pf346 zebrafish as a novel model to study Gata2 haploinsufficiency syndromes. *HemaSphere* 3 124–125.

[B66] Gitler A. D. Zhu Y. Ismat F. A. Lu M. M. Yamauchi Y. Parada L. F. (2003). Nf1 has an essential role in endothelial cells. *Nat. Genet.* 33 75–79. 10.1038/ng1059 12469121 PMC3079412

[B67] Gombart A. F. Hofmann W. K. Kawano S. Takeuchi S. Krug U. Kwok S. H. (2002). Mutations in the gene encoding the transcription factor CCAAT/enhancer binding protein α in myelodysplastic syndromes and acute myeloid leukemias. *Blood* 99 1332–1340. 10.1182/blood.V99.4.1332 11830484

[B68] Gore A. V. Pillay L. M. Venero Galanternik M. Weinstein B. M. (2018). The zebrafish: a fintastic model for hematopoietic development and disease. *Wiley Interdiscip. Rev. Dev. Biol.* 7:e312. 10.1002/wdev.312 29436122 PMC6785202

[B69] Gossage L. Eisen T. Maher E. R. (2015). VHL, the story of a tumour suppressor gene. *Nat. Rev. Cancer* 15 55–64. 10.1038/nrc3844 25533676

[B70] Growney J. D. Shigematsu H. Li Z. Lee B. H. Adelsperger J. Rowan R. (2005). Loss of Runx1 perturbs adult hematopoiesis and is associated with a myeloproliferative phenotype. *Blood* 106 494–504. 10.1182/blood-2004-08-3280 15784726 PMC1895175

[B71] Guerra F. Rocher A. Díaz L. Palaoro L. (2020). Wnt/Beta-catenin and EGFR/PI3K/pAKT/mTOR signaling pathways and their relation with cervical cancer. *J. Gynecol. Oncol.* 3:1035.

[B72] Guldberg P. Straten P. Ahrenkiel V. Seremet T. Kirkin A. F. Zeuthen J. (1999). Somatic mutation of the Peutz-Jeghers syndrome gene, LKB1/STK11, in malignant melanoma. *Oncogene* 18 1777–1780. 10.1038/sj.onc.1202486 10208439

[B73] Guo H.-F. Tong J. Hannan F. Luo L. Zhong Y. (2000). A neurofibromatosis-1-regulated pathway is required for learning in *Drosophila*. *Nature* 403 895–898. 10.1038/35002593 10706287

[B74] Hahn C. N. Chong C. E. Carmichael C. L. Wilkins E. J. Brautigan P. J. Li X. C. (2011). Heritable GATA2 mutations associated with familial myelodysplastic syndrome and acute myeloid leukemia. *Nat. Genet.* 43 1012–1019. 10.1038/ng.913 21892162 PMC3184204

[B75] Hainaut P. Pfeifer G. P. (2016). Somatic TP53 mutations in the era of genome sequencing. *Cold Spring Harb. Perspect. Med.* 6:a026179. 10.1101/cshperspect.a026179 27503997 PMC5088513

[B76] Hammond S. M. (2015). An overview of microRNAs. *Adv. Drug Deliv. Rev.* 87 3–14. 10.1016/j.addr.2015.05.001 25979468 PMC4504744

[B77] Haramis A.-P. G. Hurlstone A. van der Velden Y. Begthel H. van den Born M. Offerhaus G. J. A. (2006). Adenomatous polyposis coli-deficient zebrafish are susceptible to digestive tract neoplasia. *EMBO Rep.* 7 444–449. 10.1038/sj.embor.7400638 16439994 PMC1456916

[B78] Hardie D. G. (2007). AMP-activated/SNF1 protein kinases: conserved guardians of cellular energy. *Nat. Rev. Mol. Cell Biol.* 8 774–785. 10.1038/nrm2249 17712357

[B79] Hearle N. Schumacher V. Menko F. Olschwang S. Boardman L. Gille J. (2006). Frequency and Spectrum of Cancers in the Peutz-Jeghers Syndrome. *Clin. Cancer Res.* 12 3209–3215. 10.1158/1078-0432.CCR-06-0083 16707622

[B80] Hegedus B. Dasgupta B. Shin J. E. Emnett R. J. Hart-mahon E. K. Elghazi L. (2007). Article neurofibromatosis-1 regulates neuronal and glial cell differentiation from neuroglial progenitors in vivo by Both cAMP- and Ras-dependent mechanisms. *Cell Stem Cell* 1 443–457. 10.1016/j.stem.2007.07.008 18371380

[B81] Hemminki A. Markie D. Tomlinson I. Avizienyte E. Roth S. Loukola A. (1998). A serine/threonine kinase gene defective in Peutz–Jeghers syndrome. *Nature* 391 184–187. 10.1038/34432 9428765

[B82] Henriques C. M. Carneiro M. C. Tenente I. M. Jacinto A. Ferreira M. G. (2013). Telomerase is required for zebrafish lifespan. *PLoS Genet.* 9:e1003214. 10.1371/journal.pgen.1003214 23349637 PMC3547866

[B83] Hisada M. Garber J. E. Fung C. Y. Joseph F. Li F. P. (1998). Multiple primary cancers in families with Li-Fraumeni syndrome. *J. Natl. Cancer Inst.* 90 606–611. 9554443 10.1093/jnci/90.8.606

[B84] Hockings C. Deaner V. Hoade Y. Dace P. Lubin A. Payne E. (2018). A zebrafish model of cooperating C and N terminal CEBPA mutations reveals defects in early myelopoeisis and HSPCs leading to leukaemogenesis. *Blood* 132:1343. 10.1182/blood-2018-99-119336

[B85] Holtkamp N. Mautner V. F. Friedrich R. E. Harder A. Hartmann C. Theallier-Janko A. (2004). Differentially expressed genes in neurofibromatosis 1-associated neurofibromas and malignant peripheral nerve sheath tumors. *Acta Neuropathol.* 107 159–168. 10.1007/s00401-003-0797-8 14673600

[B86] Hsu A. P. Johnson K. D. Falcone E. L. Sanalkumar R. Sanchez L. Hickstein D. D. (2013). GATA2 haploinsufficiency caused by mutations in a conserved intronic element leads to MonoMAC syndrome. *Blood* 121 3830–3837. 10.1182/blood-2012-08-452763 23502222 PMC3650705

[B87] Hsu A. P. Sampaio E. P. Khan J. Calvo K. R. Lemieux J. E. Patel S. Y. (2011). Mutations in GATA2 are associated with the autosomal dominant and sporadic monocytopenia and mycobacterial infection (MonoMAC) syndrome. *Blood* 118 2653–2655. 10.1182/blood-2011-05-356352 21670465 PMC3172785

[B88] Huang K. Mashl R. J. Wu Y. Ritter D. I. Wang J. Oh C. (2018). Pathogenic germline variants in 10,389 adult cancers. *Cell* 173 355.e14–370.e14. 10.1016/j.cell.2018.03.039 29625052 PMC5949147

[B89] Hurlstone A. F. L. Haramis A.-P. G. Wienholds E. Begthel H. Korving J. van Eeden F. (2003). The Wnt/β-catenin pathway regulates cardiac valve formation. *Nature* 425 633–637. 10.1038/nature02028 14534590

[B90] Ichikawa M. Asai T. Saito T. Yamamoto G. Seo S. Yamazaki I. (2004). AML-1 is required for megakaryocytic maturation and lymphocytic differentiation, but not for maintenance of hematopoietic stem cells in adult hematopoiesis. *Nat. Med.* 10 299–304. 10.1038/nm997 14966519

[B91] Ignatius M. S. Hayes M. N. Moore F. E. Tang Q. Garcia S. P. Blackburn P. R. (2018). tp53 Deficiency causes a wide tumor spectrum and increases embryonal rhabdomyosarcoma metastasis in zebrafish. *Elife* 7:e37202. 10.7554/eLife.37202.001 PMC6128690 30192230

[B92] Ijsselsteijn R. Jansen J. G. de Wind N. (2020). DNA mismatch repair-dependent DNA damage responses and cancer. *DNA Repair* 93:102923. 10.1016/j.dnarep.2020.102923 33087264

[B93] Imai K. Morio T. Zhu Y. Jin Y. Itoh S. Kajiwara M. (2004). Clinical course of patients with WASP gene mutations. *Blood* 103 456–464. 10.1182/blood-2003-05-1480 12969986

[B94] Imamura S. Kishi S. (2005). Molecular cloning and functional characterization of zebrafish ATM. *Int. J. Biochem. Cell Biol.* 37 1105–1116. 10.1016/j.biocel.2004.10.015 15743681

[B95] Inoki K. Ouyang H. Zhu T. Lindvall C. Wang Y. Zhang X. (2006). TSC2 integrates wnt and energy signals via a coordinated phosphorylation by AMPK and GSK3 to regulate cell growth. *Cell* 126 955–968. 10.1016/j.cell.2006.06.055 16959574

[B96] Jette C. Peterson P. W. Sandoval I. T. Manos E. J. Hadley E. Ireland C. M. (2004). The tumor suppressor adenomatous polyposis coli and caudal related homeodomain protein regulate expression of retinol dehydrogenase L. *J. Biol. Chem.* 279 34397–34405. 10.1074/jbc.M314021200 15190067

[B97] Jopling C. van Geemen D. den Hertog J. (2007). Shp2 knockdown and Noonan/LEOPARD mutant Shp2-Induced gastrulation defects. *PLoS Genet.* 3, e225–e225. 10.1371/journal.pgen.0030225 18159945 PMC2151089

[B98] Ji H. Ramsey M. R. Hayes D. N. Fan C. McNamara K. Kozlowski P. (2007). LKB1 modulates lung cancer differentiation and metastasis. *Nature* 448 807–810. 10.1038/nature06030 17676035

[B99] Jiang N. Dai Q. Su X. Fu J. Feng X. Peng J. (2020). Role of PI3K/AKT pathway in cancer: the framework of malignant behavior. *Mol. Biol. Rep.* 47 4587–4629. 10.1007/s11033-020-05435-1 32333246 PMC7295848

[B100] Jin H. Li L. Xu J. Zhen F. Zhu L. Liu P. P. (2012). Runx1 regulates embryonic myeloid fate choice in zebrafish through a negative feedback loop inhibiting Pu.1 expression. *Blood* 119 5239–5249. 10.1182/blood-2011-12-398362 22493295 PMC3369614

[B101] Jin Y. Mazza C. Christie J. R. Giliani S. Fiorini M. Mella P. (2004). Mutations of the Wiskott-Aldrich Syndrome Protein (WASP): hotspots, effect on transcription, and translation and phenotype/genotype correlation. *Blood* 104 4010–4019. 10.1182/blood-2003-05-1592 15284122

[B102] Jing L. Zon L. I. (2011). Zebrafish as a model for normal and malignant hematopoiesis. *Dis. Model. Mech.* 4 433–438. 10.1242/dmm.006791 21708900 PMC3124047

[B103] Jones R. A. Feng Y. Worth A. J. Thrasher A. J. Burns S. O. Martin P. (2013). Modelling of human Wiskott-Aldrich syndrome protein mutants in zebrafish larvae using in vivo live imaging. *J. Cell Sci.* 126 4077–4084. 10.1242/jcs.128728 23868979 PMC3772384

[B104] Jongmans M. C. J. van der Burgt I. Hoogerbrugge P. M. Noordam K. Yntema H. G. Nillesen W. M. (2011). Cancer risk in patients with Noonan syndrome carrying a PTPN11 mutation. *Eur. J. Hum. Genet.* 19 870–874. 10.1038/ejhg.2011.37 21407260 PMC3172922

[B105] Kaidanovich-Beilin O. Woodgett J. R. (2011). GSK-3: functional insights from cell biology and animal models. *Front. Mol. Neurosci.* 4:40. 10.3389/fnmol.2011.00040 22110425 PMC3217193

[B106] Kanda M. Sadakari Y. Borges M. Topazian M. Farrell J. Syngal S. (2013). Mutant TP53 in duodenal samples of pancreatic juice from patients with pancreatic cancer or high-grade dysplasia. *Clin. Gastroenterol. Hepatol.* 11 719.e5–730.e5. 10.1016/j.cgh.2012.11.016 23200980 PMC3600161

[B107] Keszei M. Record J. Kritikou J. S. Wurzer H. Geyer C. Thiemann M. (2018). Constitutive activation of WASp in X-linked neutropenia renders neutrophils hyperactive. *J. Clin. Invest.* 128 4115–4131. 10.1172/JCI64772 30124469 PMC6118594

[B108] Ki D. H. He S. Rodig S. Look A. T. (2017). Overexpression of PDGFRA cooperates with loss of NF1 and p53 to accelerate the molecular pathogenesis of malignant peripheral nerve sheath tumors. *Oncogene* 36 1058–1068. 10.1038/onc.2016.269 27477693 PMC5332555

[B109] Ki D. H. Oppel F. Durbin A. D. Look A. T. (2019). Mechanisms underlying synergy between DNA topoisomerase I-targeted drugs and mTOR kinase inhibitors in NF1-associated malignant peripheral nerve sheath tumors. *Oncogene* 38 6585–6598. 10.1038/s41388-019-0965-5 31444410

[B110] Kikuchi H. Miyazaki S. Setohuchi T. Hiramatsu Y. Ohta M. Kamiya K. (2012). Rapid relapse after resection of a sunitinib-resistant gastrointestinal stromal tumor harboring a secondary mutation in exon 13 of the c-KIT gene. *Anticancer Res.* 32 4105–4109. 22993368

[B111] Kim H. R. Santhakumar K. Markham E. Baldera D. Greenald D. Bryant H. E. (2020). Investigation of the role of VHL-HIF signaling in DNA repair and apoptosis in zebrafish. *Oncotarget* 11 1109–1130. 10.18632/oncotarget.27521 32284789 PMC7138166

[B112] Kim S.-H. Kowalski M. L. Carson R. P. Bridges L. R. Ess K. C. (2013). Heterozygous inactivation of tsc2 enhances tumorigenesis in p53 mutant zebrafish. *Dis. Model. Mech.* 6 925–933. 10.1242/dmm.011494 23580196 PMC3701212

[B113] Kim S.-H. Speirs C. K. Solnica-Krezel L. Ess K. C. (2011). Zebrafish model of tuberous sclerosis complex reveals cell-autonomous and non-cell-autonomous functions of mutant tuberin. *Dis. Model. Mech.* 4 255–267. 10.1242/dmm.005587 20959633 PMC3046101

[B114] Kipling D. Cooke H. J. (1990). Hypervariable ultra-long telomeres in mice. *Nature* 347 400–402. 10.1038/347400a0 2170845

[B115] Kiss T. Fayet-Lebaron E. Jády B. E. (2010). Box H/ACA small ribonucleoproteins. *Mol. Cell* 37 597–606. 10.1016/j.molcel.2010.01.032 20227365

[B116] Knight S. W. Heiss N. S. Vulliamy T. J. Aalfs C. M. McMahon C. Richmond P. (1999). Unexplained aplastic anaemia, immunodeficiency, and cerebellar hypoplasia (Hoyeraal-Hreidarsson syndrome) due to mutations in the dyskeratosis congenita gene. DKC1. *Br. J. Haematol.* 107 335–339. 10.1046/j.1365-2141.1999.01690.x 10583221

[B117] Kontaridis M. I. Swanson K. D. David F. S. Barford D. Neel B. G. (2006). PTPN11 (Shp2) mutations in LEOPARD syndrome have dominant negative, not activating, effects. *J. Biol. Chem.* 281 6785–6792. 10.1074/jbc.M513068200 16377799

[B118] Kratz C. P. Schubbert S. Bollag G. Niemeyer C. M. Shannon K. M. Zenker M. (2006). Germline mutations in components of the ras signaling pathway in noonan syndrome and related disorders. *Cell Cycle* 5 1607–1611. 10.4161/cc.5.15.3128 16921267

[B119] Kroeger P. T. Drummond B. E. Miceli R. McKernan M. Gerlach G. F. Marra A. N. (2017). The zebrafish kidney mutant zeppelin reveals that brca2/fancd1 is essential for pronephros development. *Dev. Biol.* 428 148–163. 10.1016/j.ydbio.2017.05.025 28579318 PMC5571230

[B120] Kuang X. Liu C. Fang J. Ma W. Zhang J. Cui S. (2016). The tumor suppressor gene lkb1 is essential for glucose homeostasis during zebrafish early development. *FEBS Lett.* 2076–2085. 10.1002/1873-3468.12237 27264935

[B121] Kullmann L. Krahn M. P. (2018). Controlling the master—upstream regulation of the tumor suppressor LKB1. *Oncogene* 37 3045–3057. 10.1038/s41388-018-0145-z 29540834

[B122] Kutler D. I. Singh B. Satagopan J. Batish S. D. Berwick M. Giampietro P. F. (2003). A 20-year perspective on the International Fanconi Anemia Registry (IFAR). *Blood* 101 1249–1256. 10.1182/blood-2002-07-2170 12393516

[B123] Latger-Cannard V. Philippe C. Bouquet A. Baccini V. Alessi M. C. Ankri A. (2016). Haematological spectrum and genotype-phenotype correlations in nine unrelated families with RUNX1 mutations from the French network on inherited platelet disorders. *Orphanet J. Rare Dis.* 11:49. 10.1186/s13023-016-0432-0 27112265 PMC4845427

[B124] Lavalou P. Eckert H. Damy L. Constanty F. Majello S. Bitetti A. (2019). Strategies for genetic inactivation of long noncoding RNAs in zebrafish. *RNA* 25 897–904. 10.1261/rna.069484.118 31043511 PMC6633201

[B125] Leal M. C. Feitsma H. Cuppen E. França L. R. Schulz R. W. (2008). Completion of meiosis in male zebrafish (*Danio rerio*) despite lack of DNA mismatch repair gene mlh1. *Cell Tissue Res.* 332 133–139. 10.1007/s00441-007-0550-z 18247060 PMC2668577

[B126] Lee W. Teckie S. Wiesner T. Ran L. Prieto Granada C. N. Lin M. (2014). PRC2 is recurrently inactivated through EED or SUZ12 loss in malignant peripheral nerve sheath tumors. *Nat. Genet.* 46 1227–1232. 10.1038/ng.3095 25240281 PMC4249650

[B127] Li F. P. Fraumeni J. F. Mulvihill J. J. Blattner W. A. Dreyfus M. G. Tucker M. A. (1988). A cancer family syndrome in twenty-four kindreds. *Cancer Res.* 48 5358–5362. 3409256

[B128] Li J. Jørgensen S. F. Maggadottir S. M. Bakay M. Warnatz K. Glessner J. (2015). Association of CLEC16A with human common variable immunodeficiency disorder and role in murine B cells. *Nat. Commun.* 6:6804. 10.1038/ncomms7804 25891430 PMC4444044

[B129] Lim C.-H. Chong S.-W. Jiang Y.-J. (2009). Udu deficiency activates DNA damage checkpoint. *Mol. Biol. Cell* 20 4183–4193. 10.1091/mbc.E09 19656853 PMC2754932

[B130] Lin Q. Zhang Y. Zhou R. Zheng Y. Zhao L. Huang M. (2017). Establishment of a congenital amegakaryocytic thrombocytopenia model and a thrombocyte-specific reporter line in zebrafish. *Leukemia* 31 1206–1216. 10.1038/leu.2016.320 27811851

[B131] Liu K. Petree C. Requena T. Varshney P. Varshney G. K. (2019). Expanding the CRISPR toolbox in zebrafish for studying development and disease. *Front. Cell Dev. Biol.* 7:13. 10.3389/fcell.2019.00013 30886848 PMC6409501

[B132] Liu T. X. Rhodes J. Deng M. Hsu K. Radomska H. S. Kanki J. P. (2007). Dominant-interfering C/EBPα stimulates primitive erythropoiesis in zebrafish. *Exp. Hematol.* 35 230–239. 10.1016/j.exphem.2006.10.008 17258072 PMC2967023

[B133] Liu W. Palovcak A. Li F. Zafar A. Yuan F. Zhang Y. (2020). Fanconi anemia pathway as a prospective target for cancer intervention. *Cell Biosci.* 10:39. 10.1186/s13578-020-00401-7 32190289 PMC7075017

[B134] Logan C. Y. Nusse R. (2004). The Wnt signaling pathway in development and disease. *Annu. Rev. Cell Dev. Biol.* 20 781–810. 10.1146/annurev.cellbio.20.010403.113126 15473860

[B135] Lu C. Xie M. Wendl M. C. Wang J. McLellan M. D. Leiserson M. D. M. (2015). Patterns and functional implications of rare germline variants across 12 cancer types. *Nat. Commun.* 6:10086. 10.1038/ncomms10086 26689913 PMC4703835

[B136] Lutskiy M. I. Rosen F. S. Remold-O’Donnell E. (2005). Genotype-proteotype linkage in the wiskott-aldrich syndrome. *J. Immunol.* 175 1329–1336. 10.4049/jimmunol.175.2.1329 16002738

[B137] Lyle C. L. Belghasem M. Chitalia V. C. (2019). c-Cbl: an important regulator and a target in angiogenesis and tumorigenesis. *Cells* 8:498. 10.3390/cells8050498 31126146 PMC6563115

[B138] MacDonald B. T. Tamai K. He X. (2009). Wnt/β-catenin signaling: components, mechanisms, and diseases. *Dev. Cell* 17 9–26. 10.1016/j.devcel.2009.06.016 19619488 PMC2861485

[B139] Malaquias A. C. Jorge A. A. L. (2014). *Developmental Syndromes of Ras/MAPK Pathway Dysregulation in eLS.* Chichester: John Wiley & Sons, Ltd, 10.1002/9780470015902.a0021426

[B140] Malkin D. (1993). p53 and the Li-Fraumeni syndrome. *Cancer Genet. Cytogenet.* 66 83–92. 10.1016/0165-4608(93)90233-c 8500106

[B141] Malkin D. (2011). Li-fraumeni syndrome. *Genes Cancer* 2 475–484. 10.1177/1947601911413466 21779515 PMC3135649

[B142] Mans L. A. Querol Cano L. van Pelt J. Giardoglou P. Keune W.-J. Haramis A.-P. G. (2017). The tumor suppressor LKB1 regulates starvation-induced autophagy under systemic metabolic stress. *Sci. Rep.* 7:7327. 10.1038/s41598-017-07116-9 28779098 PMC5544676

[B143] Mantripragada K. K. Spurlock G. Kluwe L. Chuzhanova N. Ferner R. E. Frayling I. M. (2008). High-resolution DNA copy number profiling of malignant peripheral nerve sheath tumors using targeted microarray-based comparative genomic hybridization. *Clin. Cancer Res.* 14 1015–1024. 10.1158/1078-0432.CCR-07-1305 18281533

[B144] Marinaccio C. Suraneni P. K. Celik H. Volk A. Wen J. Q. Ling T. (2020). Loss of LKB1/STK11 facilitates leukemic progression of the myeloproliferative neoplasms. *Blood* 136 1–1. 10.1182/blood-2020-14055732430499

[B145] Martínez-Quintana E. Rodríguez-González F. (2013). RASopathies: from noonan to LEOPARD syndrome. *Rev. Española Cardiol.* 66 756–757. 10.1016/j.rec.2013.05.005 24773692

[B146] Martin-Orozco E. Sanchez-Fernandez A. Ortiz-Parra I. Ayala-San Nicolas M. (2019). WNT signaling in tumors: the way to evade drugs and immunity. *Front. Immunol.* 10:2854. 10.3389/fimmu.2019.02854 31921125 PMC6934036

[B147] Matsumoto S. Iwakawa R. Takahashi K. Kohno T. Nakanishi Y. Matsuno Y. (2007). Prevalence and specificity of LKB1 genetic alterations in lung cancers. *Oncogene* 26 5911–5918. 10.1038/sj.onc.1210418 17384680 PMC3457639

[B148] McReynolds L. J. Savage S. A. (2017). Pediatric leukemia susceptibility disorders: manifestations and management. *Hematology* 2017 242–250. 10.1182/asheducation-2017.1.242 29222262 PMC6142612

[B149] Metcalf J. L. Bradshaw P. S. Komosa M. Greer S. N. Stephen Meyn M. Ohh M. (2014). K63-Ubiquitylation of VHL by SOCS1 mediates DNA double-strand break repair. *Oncogene* 33 1055–1065. 10.1038/onc.2013.22 23455319

[B150] Miura H. Quadros R. M. Gurumurthy C. B. Ohtsuka M. (2018). Easi-CRISPR for creating knock-in and conditional knockout mouse models using long ssDNA donors. *Nat. Protoc.* 13 195–215. 10.1038/nprot.2017.153 29266098 PMC6058056

[B151] Molina J. R. Adjei A. A. (2006). The Ras/Raf/MAPK Pathway. *J. Thorac. Oncol.* 1 7–9. 10.1016/s1556-0864(15)31506-9 17409820

[B152] Moser A. Pitot H. Dove W. (1990). A dominant mutation that predisposes to multiple intestinal neoplasia in the mouse. *Science* 247 322–324. 10.1126/science.2296722 2296722

[B153] Mukherjee S. Ridgeway A. D. Lamb D. J. (2010). DNA mismatch repair and infertility. *Curr. Opin. Urol.* 20 525–532. 10.1097/MOU.0b013e32833f1c21 20852424 PMC3079360

[B154] Mullany L. K. Wong K.-K. Marciano D. C. Katsonis P. King-Crane E. R. Ren Y. A. (2015). Specific TP53 mutants overrepresented in ovarian cancer impact CNV, TP53 activity, responses to Nutlin-3a, and cell survival. *Neoplasia* 17 789–803. 10.1016/j.neo.2015.10.003 26585234 PMC4656807

[B155] Nadauld L. D. Sandoval I. T. Chidester S. Yost H. J. Jones D. A. (2004). Adenomatous polyposis coli control of retinoic acid biosynthesis is critical for zebrafish intestinal development and differentiation. *J. Biol. Chem.* 279 51581–51589. 10.1074/JBC.M408830200 15358764

[B156] Nadauld L. D. Shelton D. N. Chidester S. Yost H. J. Jones D. A. (2005). The zebrafish retinol dehydrogenase, rdh1l, Is essential for intestinal development and is regulated by the tumor suppressor adenomatous polyposis coli. *J. Biol. Chem.* 280 30490–30495. 10.1074/JBC.M504973200 15967793

[B157] Neel B. G. Gu H. Pao L. (2003). The ‘Shp’ing news: SH2 domain-containing tyrosine phosphatases in cell signaling. *Trends Biochem. Sci.* 28 284–293. 10.1016/S0968-0004(03)00091-4 12826400

[B158] Nerlov C. (2004). C/EBPα mutations in acute myeloid leukaemias. *Nat. Rev. Cancer* 4 394–400. 10.1038/nrc1363 15122210

[B159] Niemeyer C. M. Kang M. W. Shin D. H. Furlan I. Erlacher M. Bunin N. J. (2010). Germline CBL mutations cause developmental abnormalities and predispose to juvenile myelomonocytic leukemia. *Nat. Genet.* 42 794–800. 10.1038/ng.641 20694012 PMC4297285

[B160] Nye J. Melters D. P. Dalal Y. (2018). The art of War: harnessing the epigenome against cancer. *F1000Res.* 7:141. 10.12688/f1000research.12833.1 29479426 PMC5801563

[B161] Okuda T. Van Deursen J. Hiebert S. W. Grosveld G. Downing J. R. (1996). AML1, the target of multiple chromosomal translocations in human leukemia, is essential for normal fetal liver hematopoiesis. *Cell* 84 321–330. 10.1016/S0092-8674(00)80986-1 8565077

[B162] Oppel F. Tao T. Shi H. Ross K. N. Zimmerman M. W. He S. (2019). Loss of atrx cooperates with p53-deficiency to promote the development of sarcomas and other malignancies. *PLoS Genet.* 15:e1008039. 10.1371/journal.pgen.1008039 30970016 PMC6476535

[B163] Oshima M. Oshima H. Kitagawa K. Kobayashi M. Itakura C. Taketo M. (1995). Loss of Apc heterozygosity and abnormal tissue building in nascent intestinal polyps in mice carrying a truncated Apc gene. *Proc. Natl. Acad. Sci. U.S.A.* 92 4482–4486. 10.1073/pnas.92.10.4482 7753829 PMC41968

[B164] Oyarbide U. Shah A. N. Amaya-Mejia W. Snyderman M. Kell M. J. Allende D. S. (2020). Loss of Sbds in zebrafish leads to neutropenia and pancreas and liver atrophy. *JCI Insight* 5:e134309. 10.1172/jci.insight.134309 32759502 PMC7526460

[B165] Oyarbide U. Topczewski J. Corey S. J. (2019). Peering through zebrafish to understand inherited bone marrow failure syndromes. *Haematologica* 104 13–24. 10.3324/haematol.2018.196105 30573510 PMC6312012

[B166] Pabst T. Mueller B. U. Zhang P. Radomska H. S. Narravula S. Schnittger S. (2001). Dominant-negative mutations of CEBPA, encoding CCAAT/enhancer binding protein-α (C/EBPα), in acute myeloid leukemia. *Nat. Genet.* 27 263–270. 10.1038/85820 11242107

[B167] Parant J. M. George S. A. Holden J. A. Yost H. J. (2010). Genetic modeling of Li-Fraumeni syndrome in zebrafish. *Dis. Model. Mech.* 3 45–56. 10.1242/dmm.003749 20075382 PMC2806900

[B168] Peeters K. Stassen J.-M. Collen D. Van Geet C. Freson K. (2008). Emerging treatments for thrombocytopenia: increasing platelet production. *Drug Discov. Today* 13 798–806. 10.1016/j.drudis.2008.06.002 18602017

[B169] Peng X. Dong M. Ma L. Jia X. E. Mao J. Jin C. (2015). A point mutation of zebrafish c-cbl gene in the ring finger domain produces a phenotype mimicking human myeloproliferative disease. *Leukemia* 29 2355–2365. 10.1038/leu.2015.154 26104663 PMC6022398

[B170] Pereboom T. C. Van Weele L. J. Bondt A. MacInnes A. W. (2011). A zebrafish model of dyskeratosis congenita reveals hematopoietic stem cell formation failure resulting from ribosomal protein-mediated p53 stabilization. *Blood* 118 5458–5465. 10.1182/blood-2011-04-351460 21921046

[B171] Perez B. Mechinaud F. Galambrun C. Ben Romdhane N. Isidor B. Philip N. (2010). Germline mutations of the CBL gene define a new genetic syndrome with predisposition to juvenile myelomonocytic leukaemia. *J. Med. Genet.* 47 686–691. 10.1136/jmg.2010.076836 20543203

[B172] Phelps R. A. Chidester S. Dehghanizadeh S. Phelps J. Sandoval I. T. Rai K. (2009). A two-step model for colon adenoma initiation and progression caused by APC loss. *Cell* 137 623–634. 10.1016/j.cell.2009.02.037 19450512 PMC2706149

[B173] Porta C. Paglino C. Mosca A. (2014). Targeting PI3K/Akt/mTOR signaling in cancer. *Front. Oncol.* 4:64. 10.3389/fonc.2014.00064 24782981 PMC3995050

[B174] Preudhomme C. Renneville A. Bourdon V. Philippe N. Roche-Lestienne C. Boissel N. (2009). High frequency of RUNX1 biallelic alteration in acute myeloid leukemia secondary to familial platelet disorder. *Blood* 113 5583–5587. 10.1182/blood-2008-07-168260 19357396

[B175] Prior I. A. Lewis P. D. Mattos C. (2012). A comprehensive survey of ras mutations in cancer. *Cancer Res.* 72 2457–2467. 10.1158/0008-5472.CAN-11-2612 22589270 PMC3354961

[B176] Prossomariti A. Piazzi G. Alquati C. Ricciardiello L. (2020). Are Wnt/β-Catenin and PI3K/AKT/mTORC1 distinct pathways in colorectal cancer? *Cell. Mol. Gastroenterol. Hepatol.* 10 491–506. 10.1016/j.jcmgh.2020.04.007 32334125 PMC7369353

[B177] Provost E. Wehner K. A. Zhong X. Ashar F. Nguyen E. Green R. (2012). Ribosomal biogenesis genes play an essential and p53- independent role in zebrafish pancreas development. *Development* 139 3232–3241. 10.1242/dev.077107 22872088 PMC3413166

[B178] Prykhozhij S. V. Berman J. N. (2018). Zebrafish knock-ins swim into the mainstream. *Dis. Model. Mech.* 11:dmm037515. 10.1242/dmm.037515 30366936 PMC6215421

[B179] Prykhozhij S. V. Caceres L. Berman J. N. (2018a). New developments in CRISPR/Cas-based functional genomics and their implications for research using Zebrafish. *Curr. Gene Ther.* 17 286–300. 10.2174/1566523217666171121164132 29173171

[B180] Prykhozhij S. V. Fuller C. Steele S. L. Veinotte C. J. Razaghi B. Robitaille J. M. (2018b). Optimized knock-in of point mutations in zebrafish using CRISPR/Cas9. *Nucleic Acids Res.* 46:e102. 10.1093/nar/gky512 29905858 PMC6158492

[B181] Prykhozhij S. V. Cordeiro-Santanach A. Caceres L. Berman J. N. (2020). “Genome editing in zebrafish using high-fidelity Cas9 nucleases: choosing the right nuclease for the task. *Methods Mol. Biol.* 2115 385–405. 10.1007/978-1-0716-0290-4_21 32006412

[B182] Qu Y. Gharbi N. Yuan X. Olsen J. R. Blicher P. Dalhus B. (2016). Axitinib blocks Wnt/β-catenin signaling and directs asymmetric cell division in cancer. *Proc. Natl. Acad. Sci. U. S. A.* 113 9339–9344. 10.1073/pnas.1604520113 27482107 PMC4995957

[B183] Rabinowitz J. D. White E. (2010). Autophagy and Metabolism. *Science* 330 1344–1348. 10.1126/science.1193497 21127245 PMC3010857

[B184] Raby L. Völkel P. Le Bourhis X. Angrand P. O. (2020). Genetic engineering of zebrafish in cancer research. *Cancers* 12:2168. 10.3390/cancers12082168 32759814 PMC7464884

[B185] Raghunandan M. Yeo J. E. Walter R. Saito K. Harvey A. J. Ittershagen S. (2020). Functional cross talk between the Fanconi anemia and ATRX/DAXX histone chaperone pathways promotes replication fork recovery. *Hum. Mol. Genet.* 29 1083–1095. 10.1093/hmg/ddz250 31628488 PMC7206856

[B186] Rahman N. (2014). Realizing the promise of cancer predisposition genes. *Nature* 505 302–308. 10.1038/nature12981 24429628 PMC4975511

[B187] Rai K. Sarkar S. Broadbent T. J. Voas M. Grossmann K. F. Nadauld L. D. (2010). DNA demethylase activity maintains intestinal cells in an undifferentiated state following loss of APC. *Cell* 142, 930–942. 10.1016/j.cell.2010.08.030 20850014 PMC2943938

[B188] Ramanagoudr-Bhojappa R. Carrington B. Ramaswami M. Bishop K. Robbins G. M. Jones M. P. (2018). Multiplexed CRISPR/Cas9-mediated knockout of 19 Fanconi anemia pathway genes in zebrafish revealed their roles in growth, sexual development and fertility. *PLoS Genet.* 14:e1007821. 10.1371/journal.pgen.1007821 30540754 PMC6328202

[B189] Rasighaemi P. Basheer F. Liongue C. Ward A. C. (2015). Zebrafish as a model for leukemia and other hematopoietic disorders. *J. Hematol. Oncol.* 8:29. 10.1186/s13045-015-0126-4 25884214 PMC4389495

[B190] Rasnic R. Linial N. Linial M. (2020). Expanding cancer predisposition genes with ultra-rare cancer-exclusive human variations. *Sci. Rep.* 10:13462. 10.1038/s41598-020-70494-0 32778766 PMC7418036

[B191] Ries S. Biederer C. Woods D. Shifman O. Shirasawa S. Sasazuki T. (2000). Opposing effects of Ras on p53. *Cell* 103 321–330. 10.1016/S0092-8674(00)00123-9 11057904

[B192] Ripperger T. Steinemann D. Göhring G. Finke J. Niemeyer C. M. Strahm B. (2009). A novel pedigree with heterozygous germline RUNX1 mutation causing familial MDS-related AML: can these families serve as a multistep model for leukemic transformation? *Leukemia* 23 1364–1366. 10.1038/leu.2009.87 19387465

[B193] Robertson J. C. Jorcyk C. L. Oxford J. T. (2018). DICER1 syndrome: DICER1 mutations in rare cancers. *Cancers* 10:143. 10.3390/cancers10050143 29762508 PMC5977116

[B194] Rodríguez-Marí A. Cañestro C. BreMiller R. A. Nguyen-Johnson A. Asakawa K. Kawakami K. (2010). Sex reversal in zebrafish fancl mutants is caused by Tp53-mediated germ cell apoptosis. *PLoS Genet.* 6:e1001034. 10.1371/journal.pgen.1001034 20661450 PMC2908690

[B195] Rodríguez-Marí A. Wilson C. Titus T. A. Cañestro C. BreMiller R. A. Yan Y. L. (2011). Roles of brca2 (fancd1) in oocyte nuclear architecture, gametogenesis, gonad tumors, and genome stability in zebrafish. *PLoS Genet.* 7:e1001357. 10.1371/journal.pgen.1001357 21483806 PMC3069109

[B196] Rounioja S. Saralahti A. Rantala L. Parikka M. Henriques-Normark B. Silvennoinen O. (2012). Defense of zebrafish embryos against *Streptococcus pneumoniae* infection is dependent on the phagocytic activity of leukocytes. *Dev. Comp. Immunol.* 36 342–348. 10.1016/j.dci.2011.05.008 21658407

[B197] Sansal I. Sellers W. R. (2004). The biology and clinical relevance of the PTEN tumor suppressor pathway. *J. Clin. Oncol.* 22 2954–2963. 10.1200/JCO.2004.02.141 15254063

[B198] Santoriello C. Deflorian G. Pezzimenti F. Kawakami K. Lanfrancone L. Di Fagagna F. D. A. (2009). Expression of H-RASV12 in a zebrafish model of costello syndrome causes cellular senescence in adult proliferating cells. *Dis. Model. Mech.* 2 56–67. 10.1242/dmm.001016 19132118 PMC2615164

[B199] Sarkozy A. Digilio M. C. Dallapiccola B. (2008). Leopard syndrome. *Orphanet J. Rare Dis.* 3:13. 10.1186/1750-1172-3-13 18505544 PMC2467408

[B200] Schmit J. M. Turner D. J. Hromas R. A. Wingard J. R. Brown R. A. Li Y. (2015). Two novel RUNX1 mutations in a patient with congenital thrombocytopenia that evolved into a high grade myelodysplastic syndrome. *Leuk. Res. Rep.* 4 24–27. 10.1016/j.lrr.2015.03.002 25893166 PMC4398854

[B201] Schuhmacher A. J. Guerra C. Sauzeau V. Cañamero M. Bustelo X. R. Barbacid M. (2008). A mouse model for costello syndrome reveals an Ang II-mediated hypertensive condition. *J. Clin. Invest.* 118 2169–2179. 10.1172/JCI34385 18483625 PMC2381749

[B202] Serra-Nedelec A. D. R. Edouard T. Treguer K. Tajan M. Araki T. Dance M. (2012). Noonan syndrome-causing SHP2 mutants inhibit insulin-like growth factor 1 release via growth hormone-induced ERK hyperactivation, which contributes to short stature. *Proc. Natl. Acad. Sci. U.S.A.* 109 4257–4262. 10.1073/pnas.1119803109 22371576 PMC3306697

[B203] Shaw R. J. (2009). LKB1 and AMP-activated protein kinase control of mTOR signalling and growth. *Acta Physiol.* 196 65–80. 10.1111/j.1748-1716.2009.01972.x 19245654 PMC2760308

[B204] Shelton D. N. Sandoval I. T. Eisinger A. Chidester S. Ratnavake A. Ireland C. M. (2006). Up-regulation of CYP26A1 in adenomatous polyposis coli-deficient vertebrates via a WNT-dependent mechanism: implications for intestinal cell differentiation and colon tumor development. *Cancer Res.* 66, 7571–7577. 10.1158/0008-5472.CAN-06-1067. 16885356

[B205] Shin J. Padmanabhan A. De Groh E. D. Lee J. S. Haidar S. Dahlberg S. (2012). Zebrafish neurofibromatosis type 1 genes have redundant functions in tumorigenesis and embryonic development. *Dis. Model. Mech.* 5 881–894. 10.1242/dmm.009779 22773753 PMC3484870

[B206] Shivanna S. Harrold I. Shashar M. Meyer R. Kiang C. Francis J. (2015). The C-Cbl ubiquitin ligase regulates nuclear β-catenin and angiogenesis by its tyrosine phosphorylation mediated through the Wnt signaling pathway. *J. Biol. Chem.* 290 12537–12546. 10.1074/jbc.M114.616623 25784557 PMC4432275

[B207] Shive H. R. West R. R. Embree L. J. Azuma M. Sood R. Liu P. (2010). brca2 in zebrafish ovarian development, spermatogenesis, and tumorigenesis. *Proc. Natl. Acad. Sci. U.S.A.* 107 19350–19355. 10.1073/pnas.1011630107 20974951 PMC2984219

[B208] Shive H. R. West R. R. Embree L. J. Golden C. D. Hickstein D. D. (2014). Brca2 and Tp53 collaborate in tumorigenesis in Zebrafish. *PLoS One* 9:e87177. 10.1371/journal.pone.0087177 24489863 PMC3906131

[B209] Shlien A. Tabori U. Marshall C. R. Pienkowska M. Feuk L. Novokmet A. (2008). Excessive genomic DNA copy number variation in the Li-Fraumeni cancer predisposition syndrome. *Proc. Natl. Acad. Sci. U.S.A.* 105 11264–11269. 10.1073/pnas.0802970105 18685109 PMC2516272

[B210] Silva A. J. Frankland P. W. Marowitz Z. Friedman E. Lazlo G. Cioffi D. (1997). A mouse model for the learning and memory deficits associated with neurofibromatosis type I. *Nat. Genet.* 15 281–284. 10.1038/ng0397-281 9054942

[B211] Song W. J. Sullivan M. G. Legare R. D. Hutchings S. Tan X. Kufrin D. (1999). Haploinsufficiency of CBFA2 causes familial thrombocytopenia with propensity to develop acute myelogenous leukaemia. *Nat. Genet.* 23 166–175. 10.1038/13793 10508512

[B212] Sood R. English M. A. Belele C. L. Jin H. Bishop K. Haskins R. (2010). Development of multilineage adult hematopoiesis in the zebrafish with a runx1 truncation mutation. *Blood* 115 2806–2809. 10.1182/blood-2009-08-236729 20154212 PMC2854427

[B213] Sood R. Kamikubo Y. Liu P. (2017). Role of RUNX1 in hematological malignancies. *Blood* 129 2070–2082. 10.1182/blood-2016-10-687830 28179279 PMC5391618

[B214] Stepensky P. Chacón-Flores M. Kim K. H. Abuzaitoun O. Bautista-Santos A. Simanovsky N. (2017). Mutations in EFL1, an SBDS partner, are associated with infantile pancytopenia, exocrine pancreatic insufficiency and skeletal anomalies in a Shwachman-Diamond like syndrome. *J. Med. Genet.* 54 558–566. 10.1136/jmedgenet-2016-104366 28331068

[B215] Stewart R. A. Sanda T. Widlund H. R. Zhu S. Swanson K. D. Hurley A. D. (2010). Phosphatase-dependent and -independent functions of Shp2 in neural crest cells underlie LEOPARD syndrome pathogenesis. *Dev. Cell* 18 750–762. 10.1016/j.devcel.2010.03.009 20493809 PMC3035154

[B216] Stiff T. Tena T. C. O’Driscoll M. Jeggo P. A. Philipp M. (2016). ATR promotes cilia signalling: links to developmental impacts. *Hum. Mol. Genet.* 25 1574–1587. 10.1093/hmg/ddw034 26908596 PMC4805311

[B217] Stumpf M. Choorapoikayil S. den Hertog J. (2015). Pten function in zebrafish: anything but a fish story. *Methods* 77–78 191–196. 10.1016/j.ymeth.2014.11.002 25461815

[B218] Sullivan K. E. Mullen C. A. Blaese R. M. Winkelstein J. A. (1994). A multiinstitutional survey of the Wiskott-Aldrich syndrome. *J. Pediatr.* 125 876–885. 10.1016/S0022-3476(05)82002-5 7996359

[B219] Szklarczyk D. Franceschini A. Wyder S. Forslund K. Heller D. Huerta-Cepas J. (2015). STRING v10: protein-protein interaction networks, integrated over the tree of life. *Nucleic Acids Res.* 43 D447–D452. 10.1093/nar/gku1003 25352553 PMC4383874

[B220] Tabori U. Nanda S. Druker H. Lees J. Malkin D. (2007). Younger age of cancer initiation is associated with shorter telomere length in Li-fraumeni syndrome. *Cancer Res.* 67 1415–1418. 10.1158/0008-5472.CAN-06-3682 17308077

[B221] Tan S. Kermasson L. Hoslin A. Jaako P. Faille A. Acevedo-Arozena A. (2019). EFL1 mutations impair eIF6 release to cause Shwachman-Diamond syndrome. *Blood* 134 277–290. 10.1182/blood.2018893404 31151987 PMC6754720

[B222] Tartaglia M. Gelb B. D. Zenker M. (2011). Noonan syndrome and clinically related disorders. *Best Pract. Res. Clin. Endocrinol. Metab.* 25 161–179. 10.1016/j.beem.2010.09.002 21396583 PMC3058199

[B223] Tartaglia M. Mehler E. L. Goldberg R. Zampino G. Brunner H. G. Kremer H. (2001). Mutations in PTPN11, encoding the protein tyrosine phosphatase SHP-2, cause Noonan syndrome. *Nat. Genet.* 29 465–468. 10.1038/ng772 11704759

[B224] Tawana K. Wang J. Renneville A. Bödör C. Hills R. Loveday C. (2015). Disease evolution and outcomes in familial AML with germline CEBPA mutations. *Blood* 126 1214–1223. 10.1182/blood-2015-05-647172 26162409

[B225] Thien C. B. F. Langdon W. Y. (2001). Cbl: many adaptations to regulate protein tyrosine kinases. *Nat. Rev. Mol. Cell Biol.* 2 294–307. 10.1038/35067100 11283727

[B226] Thrasher A. J. Burns S. O. (2010). WASP: a key immunological multitasker. *Nat. Rev. Immunol.* 10 182–192. 10.1038/nri2724 20182458

[B227] Titus T. A. Yan Y. L. Wilson C. Starks A. M. Frohnmayer J. D. Bremiller R. A. (2009). The Fanconi anemia/BRCA gene network in zebrafish: embryonic expression and comparative genomics. *Mutat. Res. Fundam. Mol. Mech. Mutagen.* 668 117–132. 10.1016/j.mrfmmm.2008.11.017 19101574 PMC2714409

[B228] Tong J. Hannan F. Zhu Y. Bernards A. Zhong Y. (2002). Neurofibromin regulates G protein-stimulated adenylyl cyclase activity. *Nat. Neurosci.* 5 95–96. 10.1038/nn792 11788835

[B229] Tonini G. Intagliata S. Cagli B. Segreto F. Perrone G. Onetti Muda A. (2010). Recurrent scrotal hemangiomas during treatment with Sunitinib. *J. Clin. Oncol.* 28 e737–e738. 10.1200/JCO.2010.30.4865 20823408

[B230] Tsai S.-M. Chu K.-C. Jiang Y.-J. (2020). Newly identified Gon4l/Udu-interacting proteins implicate novel functions. *Sci. Rep.* 10:14213. 10.1038/s41598-020-70855-9 32848183 PMC7449961

[B231] Tsuboi S. Meerloo J. (2007). Wiskott-aldrich syndrome protein is a key regulator of the phagocytic cup formation in macrophages. *J. Biol. Chem.* 282 34194–34203. 10.1074/jbc.M705999200 17890224

[B232] Vågbø C. B. Slupphaug G. (2020). RNA in DNA repair. *DNA Repair.* 95:102927. 10.1016/j.dnarep.2020.102927 32920299

[B233] Vakulskas C. A. Dever D. P. Rettig G. R. Turk R. Jacobi A. M. Collingwood M. A. (2018). A high-fidelity Cas9 mutant delivered as a ribonucleoprotein complex enables efficient gene editing in human hematopoietic stem and progenitor cells. *Nat. Med.* 24 1216–1224. 10.1038/s41591-018-0137-0 30082871 PMC6107069

[B234] Valvezan A. J. Huang J. Lengner C. J. Pack M. Klein P. S. (2014). Oncogenic mutations in adenomatous polyposis coli (Apc) activate mechanistic target of rapamycin complex 1 (mTORC1) in mice and zebrafish. *Dis. Model. Mech.* 7 63–71. 10.1242/dmm.012625 24092877 PMC3882049

[B235] Valvezan A. J. Zhang F. Diehl J. A. Klein P. S. (2012). Adenomatous polyposis coli (APC) regulates multiple signaling pathways by enhancing glycogen synthase kinase-3 (GSK-3) activity. *J. Biol. Chem.* 287 3823–3832. 10.1074/jbc.M111.323337 22184111 PMC3281685

[B236] Van Der Velden Y. U. Wang L. Zevenhoven J. Van Rooijen E. Van Lohuizen M. Giles R. H. (2011). The serine-threonine kinase LKB1 is essential for survival under energetic stress in zebrafish. *Proc. Natl. Acad. Sci. U. S. A.* 108 4358–4363. 10.1073/pnas.1010210108 21368212 PMC3060253

[B237] Vander Heiden M. G. Cantley L. C. Thompson C. B. (2009). Understanding the warburg effect: the metabolic requirements of cell proliferation. *Science* 324 1029–1033. 10.1126/science.1160809 19460998 PMC2849637

[B238] Venkatasubramani N. Mayer A. N. (2008). A zebrafish model for the Shwachman-Diamond Syndrome (SDS). *Pediatr. Res.* 63 348–352. 10.1203/PDR.0b013e3181659736 18356737

[B239] Vierstraete J. Willaert A. Vermassen P. Coucke P. J. Vral A. Claes K. B. M. (2017). Accurate quantification of homologous recombination in zebrafish: Brca2 deficiency as a paradigm. *Sci. Rep.* 7:16518. 10.1038/s41598-017-16725-3 29184099 PMC5705637

[B240] Vogeli K. M. Jin S.-W. Martin G. R. Stainier D. Y. R. (2006). A common progenitor for haematopoietic and endothelial lineages in the zebrafish gastrula. *Nature* 443 337–339. 10.1038/nature05045 16988712

[B241] Wang H. Xu J. Lazarovici P. Quirion R. Zheng W. (2018). cAMP Response element-binding protein (CREB): a possible signaling molecule link in the pathophysiology of Schizophrenia. *Front. Mol. Neurosci.* 11:225. 10.3389/fnmol.2018.00255 30214393 PMC6125665

[B242] Warren A. J. (2018). Molecular basis of the human ribosomopathy Shwachman-Diamond syndrome. *Adv. Biol. Regul.* 67 109–127. 10.1016/j.jbior.2017.09.002 28942353 PMC6710477

[B243] Weber A. M. Ryan A. J. (2015). ATM and ATR as therapeutic targets in cancer. *Pharmacol. Ther.* 149 124–138. 10.1016/j.pharmthera.2014.12.001 25512053

[B244] White R. M. Sessa A. Burke C. Bowman T. LeBlanc J. Ceol C. (2008). Transparent adult zebrafish as a tool for in vivo transplantation analysis. *Cell Stem Cell* 2 183–189. 10.1016/j.stem.2007.11.002 18371439 PMC2292119

[B245] Wierson W. A. Welker J. M. Almeida M. P. Mann C. M. Webster D. A. Torrie M. E. (2020). Efficient targeted integration directed by short homology in zebrafish and mammalian cells. *Elife* 9:e53968. 10.7554/eLife.53968 32412410 PMC7228771

[B246] Wingo S. N. Gallardo T. D. Akbay E. A. Liang M.-C. Contreras C. M. Boren T. (2009). Somatic LKB1 mutations promote cervical cancer progression. *PLoS One* 4:e5137. 10.1371/journal.pone.0005137 19340305 PMC2660434

[B247] Wlodarski M. W. Collin M. Horwitz M. S. (2017). GATA2 deficiency and related myeloid neoplasms. *Semin. Hematol.* 54 81–86. 10.1053/j.seminhematol.2017.05.002 28637621 PMC5650112

[B248] Wlodarski M. W. Hirabayashi S. Pastor V. Starý J. Hasle H. Masetti R. (2016). Prevalence, clinical characteristics, and prognosis of GATA2-related myelodysplastic syndromes in children and adolescents. *Blood* 127 1387–1397. 10.1182/blood-2015-09-669937 26702063

[B249] Wolman M. A. deGroh E. D. McBride S. M. Jongens T. A. Granato M. Epstein J. A. (2014). Modulation of cAMP and ras signaling pathways improves distinct behavioral deficits in a zebrafish model of neurofibromatosis type 1. *Cell Rep.* 8 1265–1270. 10.1016/j.celrep.2014.07.054 25176649 PMC5850931

[B250] Yamaguchi M. Fujimori-Tonou N. Yoshimura Y. Kishi T. Okamoto H. Masai I. (2008). Mutation of DNA primase causes extensive apoptosis of retinal neurons through the activation of DNA damage checkpoint and tumor suppressor p53. *Development* 135 1247–1257. 10.1242/dev.011015 18287205

[B251] Yang W. Klaman L. D. Chen B. Araki T. Harada H. Thomas S. M. (2006). An Shp2/SFK/Ras/Erk signaling pathway controls trophoblast stem cell survival. *Dev. Cell* 10 317–327. 10.1016/j.devcel.2006.01.002 16516835

[B252] Yuan H. Wen B. Liu X. Gao C. Yang R. Wang L. (2015). CCAAT/enhancer-binding protein α is required for hepatic outgrowth via the p53 pathway in zebrafish. *Sci. Rep.* 5:15838. 10.1038/srep15838 26511037 PMC4649991

[B253] Yuan H. Zhou J. Deng M. Zhang Y. Chen Y. Jin Y. (2011). Sumoylation of CCAAT/enhancer-binding protein α promotes the biased primitive hematopoiesis of zebrafish. *Blood* 117 7014–7020. 10.1182/blood-2010-12-325712 21596856

[B254] Zhang J. Walsh M. F. Wu G. Edmonson M. N. Gruber T. A. Easton J. (2015). Germline mutations in predisposition genes in pediatric cancer. *N. Engl. J. Med.* 373 2336–2346. 10.1056/NEJMoa1508054 26580448 PMC4734119

[B255] Zhang Y. Gao X. Saucedo L. J. Ru B. Edgar B. A. Pan D. (2003). Rheb is a direct target of the tuberous sclerosis tumour suppressor proteins. *Nat. Cell Biol*. 5 578–581. 10.1038/ncb999 12771962

[B256] Zhang Y. Morimoto K. Danilova N. Zhang B. Lin S. (2012). Zebrafish models for dyskeratosis congenita reveal critical roles of p53 activation contributing to hematopoietic defects through RNA processing. *PLoS One* 7:e30188. 10.1371/journal.pone.0030188 22299032 PMC3267717

[B257] Zhu Y. (2002). Neurofibromas in NF1: schwann cell origin and role of tumor environment. *Science* 296 920–922. 10.1126/science.1068452 11988578 PMC3024710

[B258] Zietsch J. Ziegenhagen N. Heppner F. L. Reuss D. von Deimling A. Holtkamp N. (2010). The 4q12 amplicon in malignant peripheral nerve sheath tumors: consequences on gene expression and implications for sunitinib treatment. *PLoS One* 5:e11858. 10.1371/journal.pone.0011858 20686603 PMC2912277

