# Supplementary material for: Zebrafish Cancer Predisposition Models
Source: Front Cell Dev Biol. 2021 Apr 27;9:660069. doi: 10.3389/fcell.2021.660069 (PMC8112447; doi:10.3389/fcell.2021.660069)
Supplement: Supplementary file 1 [file Table_1.docx]

**Supplementary Table 1:** A summary ofzebrafish cancer predisposition syndrome models, specifying CPGs involved, zebrafish strains, and associated phenotypes. dpf = days post-fertilization.

| **Cancer Predisposition Syndrome** | **CPG** | **Zebrafish Model** | **Tumor Types** | **Associated Phenotypes** | **Method** | **References** |
| --- | --- | --- | --- | --- | --- | --- |
| Li-Fraumeni Syndrome | *TP53* | *tp53*^N168K^ | Not reported | Resistant to apoptosisfollowing induction of DNA damage only at 37ºC | ENU | (Berghmans et al., 2005) |
|  |  | *tp53*^M214K^ | MPNSTs | Resistant to apoptosis following induction of DNA damage | ENU |  |
|  |  | *tp53*^I166T^ | Sarcomas |  | ENU | (Parant et al., 2010) |
|  |  | *tp53*^-/-^ | MPNSTs, angiosarcomas, germ cell tumors, a natural killer cell-like leukemia |  | TALEN | (Ignatius et al., 2018) |
|  |  | *tp53*^R217H^ | Not yet characterized | Not yet characterized | CRISPR | (Prykhozhij et al., 2018b) |
| Familial Adenomatous Polyposis | *APC* | *apc*^MCR/MCR^ | Intestinal adenomas in heterozygotes | Cardiac valve defect, curved body types, lack colonocyte differentiation, do not survive past 15dpf | ENU | (Hurlstone et al., 2003; Nadauld et al., 2005; Haramis et al., 2006) |
| Peutz-Jeghers Syndrome | *LKB1* | *lkb1^-/-^* | Not reported | Starvation phenotype that causes lethality by 7-8 dpf, metabolic state resembles aerobic glycolysis | ENU | (Van Der Velden et al., 2011) |
| Cowden Syndrome | *PTEN* | *ptenb­^-/-^* | Neuroepitheliomas | Phenotypically normal | ENU | (Faucherre et al., 2008) |
|  |  | *ptena*^-/-^; *ptenb*^+/-^ | Hemangiosarcomas near the eye | 10% tumor incidence | ENU | (Choorapoikayil et al., 2012) |
|  |  | *ptena*^+/-^; *ptenb*^-/-^ |  | 2% tumor incidence | Breeding |  |
|  |  | *ptena^-^*^/-^; *ptenb*^-/-^ | Not reported | Enhanced angiogenesis, lethal by 5dpf | Breeding | (Choorapoikayil et al., 2013) |
| Tuberous Sclerosis Complex | *TSC2* | *tsc2*^vu242/vu242^ | Not reported | Increased mTORC1 but lethal by 11dpf | ENU | (Kim et al., 2011) |
|  |  | *tsc2*^vu242/+^; *tp53*^M214K/M214K^ | Multiple malignancies | Increased mTORC1 and angiogenesis | Breeding | (Kim et al., 2013) |
| Noonan/LEOPARD Syndrome | *PTPN11* | *ptpn11b*^-/-^ | Not reported | Phenotypically normal | ENU | (Bonetti et al., 2014b) |
|  |  | *ptpn11a^-/-^* |  | Cardiac and pleotropic defects, lethal by 5dpf | ENU |  |
|  |  | *ptpn11a*^-/-^; *ptpn11b*^-/-^ |  |  | Breeding |  |
| *c-CBL* Mutation Associated Syndrome | *c-CBL* | *c-cbl*^H382Y^ | Not reported | Myeloproliferative phenotype, median survival of 15dpf | ENU | (Peng et al., 2015) |
| Costello Syndrome | *HRAS* | *HRAS*^G12V^ | Melanomas, gut carcinomas, hepatocarcinomas, rhabdomyosarcoma | Traits of Costello syndrome | Transgene | (Santoriello et al., 2009) |
| Neurofibromatosis Type 1 | *NF1* | *nf1a*^-/-^; *nf1b*^-/-^ | Not reported | Nervous system defects, melanophore phenotype, do not survive past 10dpf | ZFN | (Shin et al., 2012) |
|  |  | *nf1a*^+/-^; *nf1b*^-/-^; *tp53^M214K/M214K^* | High-grade gliomas and MPNSTs | Increased onset and penetrance of tumor development | Breeding |  |
|  |  | *nf1a*^+/-^; *nf1b*^-/-^; *tp53^M214K/M214K^; sox10:PDGFRA*^WT^ | MPNSTs | Nervous system defects, melanophore phenotype,decreased time to tumor onset | Breeding, Transgene | (Ki et al., 2017) |
| Dyskeratosis Congenita | *TERT* | *tert*^-/-^ | Not reported | Premature aging, reduced telomere length, decreased fertility, shortened lifespans | ENU | (Anchelin et al., 2013) |
|  | *NOP10* | hi2578 | Not reported | Fail to form HSCs, ribosomal defects, cytopenia | Retroviral insertion | (Pereboom et al., 2011) |
| Shwachman-Diamond Syndrome | *SDBS* | *sbds^nu16^* | Not reported | Growth defects, pancreatic atrophy after 15dpf, do not survive past 21dpf | CRISPR | (Oyarbide et al., 2020) |
|  |  | *sbds^nu132^* |  | Neutropenia, growth defects, pancreatic atrophy after 15dpf, do not survive past 21dpf | CRISPR |  |
| Wiskott-Aldrich Syndrome and X-linked Congenital Neutropenia | *WASp* | *wasp1*^-/-^ | Not reported | Impaired immune response | ENU | (Jones et al., 2013) |
|  |  | *wasp1*^-/-^; hWASp |  | Rescues neutrophil response to a wound site | Breeding, Transgene |  |
|  |  | *wasp1*^-/-^; hWASp^I294T^ |  | Neutropenia | Breeding, Transgene |  |
|  |  | *wasp1*^-/-^; hWASp^H246D^ |  | Rescued cell motility and recruitment | Breeding, Transgene |  |
|  |  | *wasp1*^-/-^; hWASp^Y291F^ |  | Slight improvement for neutrophil migration | Breeding, Transgene |  |
|  |  | *wasp1*^-/-^; hWASp^I294T^ |  | Hyperprotrusive neutrophils with increased velocity during wound response | Breeding, Transgene |  |
| GATA2 Deficiency Syndromes | *GATA2* | *gata2a^Δi4/Δi4^* | Not reported | Decreased blood cells in kidney marrow, immune and lymphatic defects similar to GATA2 deficiency syndromes | CRISPR | (Dobrzycki et al., 2020) |
|  |  | *gata2b*^+/-^ |  | Reduced myeloid differentiation and dysplastic myeloid cells in adults | CRISPR | (Avagyan et al., 2017; Gioacchino et al., 2019) |
| Familial AML | *CEBPA* | *moli^hkz7^* | Not reported | Defects in myeloid progenitor proliferation & differentiation | ENU | (Dai et al., 2016) |
|  |  | *cebpa^sum2^* |  |  | TALEN |  |
|  |  | *cebpa^sum3^* |  |  | TALEN |  |
|  |  | *cebpa*^Cterm/Nterm^ | Leukemic transformation between 4-6 weeks of age | Defects in mature myelocytes and monocytes | TALEN | (Hockings et al., 2018) |
|  |  | *cebpa*^Nterm/Nterm^ |  |  | TALEN |  |
| Familial Platelet Disorder with Predisposition to AML | *RUNX1* | *runx1*^W84X/W84X^ | Not reported | Reduced number of neutrophils, thrombocytes, and B-cells | ENU | (Sood et al., 2010; Jin et al., 2012; Chi et al., 2018) |
| Congenital Amegakaryocytic Thrombocytopenia | *MPL* | *mpl*^smu3^ | Not reported | Severe thrombocytopenia, reduced homeostasis and abnormal bleeding | TALEN | (Lin et al., 2017) |
| Fanconi Anemia | *FANCA* | *fanca*_hg41 | Not reported | Female-to-male sex reversal | CRISPR | (Ramanagoudr-Bhojappa et al., 2018) |
|  | *FANCB* | *fancb*_hg42 |  |  | CRISPR |  |
|  | *FANCC* | *fancc*_hg43 |  |  | CRISPR |  |
|  | *FANCD2* | *fancd2*_hg47 |  |  | CRISPR |  |
|  | *FANCE* | *fance_hg48* |  |  | CRISPR |  |
|  | *FANCF* | *fancf_hg50* |  |  | CRISPR |  |
|  | *FANCG* | *fancg_hg53* |  |  | CRISPR |  |
|  | *FANCI* | *fanci_hg54* |  |  | CRISPR |  |
|  | *FANCJ/ BRIP1* | *fancj_hg56* |  |  | CRISPR |  |
|  | *FANCJ/ BRIP1* | *fancj_hg57* |  |  | CRISPR |  |
|  | *FANCL* | *fancl_hg59* |  |  | CRISPR |  |
|  | *FANCM* | *fancm_hg60* |  |  | CRISPR |  |
|  | *FANCN/ PALB2* | *fancn_hg62* |  |  | CRISPR |  |
|  | *FANCO/ RAD51C* | *fanco_hg65* |  |  | CRISPR |  |
|  | *FANCP/ SLX4* | *fancp_hg66* |  |  | CRISPR |  |
|  | *FANCT/ UBE2T* | *fanct_hg70* |  |  | CRISPR |  |
|  | *FAAP24* | *faap24* |  |  | CRISPR |  |
|  | *FAAP100* | *faap100* |  |  | CRISPR |  |
|  | *FANCD1/BRCA2* | *fancd1*_hg45 |  |  | CRISPR |  |
|  |  | *brca2*^Q658X/Q658X^ | Testicular neoplasia | Female-to-male sex reversal | ENU | (Shive et al., 2010) |
|  |  | *brca2*^Q658X/Q658X^; tp53^+/M214K^ | MPNSTs, sarcomas, nephroblastomas | Accelerated tumorigenesis | Breeding | (Shive et al., 2010, 2014) |
|  |  | *brca2*^-/-^ | Testicular neoplasia | Female-to-male sex reversal, genome instability, male sterility | Retroviral insertion | (Rodríguez-Marí et al., 2011) |
|  |  | *brca2^-/-^; tp53*^M214K/M214K^ | Testicular and invasive ovarian tumours | Rescued ovarian development but females were sterile, male sterility, reduced apoptosis, accelerated tumorigenesis | Breeding |  |
|  |  | *zeppelin* | Not reported | Defects in kidney development, interrenal gland expansion | ENU | (Kroeger et al., 2017) |
|  | *FANCR/ RAD51* | *rad51*^-/-^ | Not reported | Female-to-male sex reversal, increased chromosomal aberrations following induction of DNA damage, size defects, decreased blood cells in adult kidney marrow | ENU | (Botthof et al., 2017) |
|  |  | *rad51*^-/-^; *tp53*^M214K/M214K^ | MPNSTs | Rescued HSPC defects and sex reversal, adult sterility, size defects, decreased time to tumor onset compared to *tp53* mutants | Breeding |  |
| CPGs not associated with a cancer predisposition syndrome | *MLH1* | *mlh1*^-/-^ | Neurofibromas, MPNSTs, PNETs, hemangiosarcomas | Males are infertile with abnormal testis histology, females are fertile but produce triploid embryos | ENU | (Feitsma et al., 2007, 2008; Leal et al., 2008) |
|  | *MSH2* | *msh2*^-/-^ |  | Microsatellite instability in mutant male progeny | ENU | (Feitsma et al., 2008) |
|  | *MSH6* | *msh6*^-/-^ |  |  | ENU |  |
|  | *ATRX* | *atrx*^-/-^ | None | Do not survive past larvae stages, erythrocytes are more spherical | CRISPR | (Oppel et al., 2019) |
|  |  | *tp53*^M214K/M214K^*;nf1b^-/-^;nf1a^+/-^;atrx^+/-^* | Epithelioid sarcomas, angiosarcomas, and rare carcinomas | Wider spectrum of tumor types, lengthening of telomeres | Breeding |  |
|  | *VHL* | *vhl^-/-^*; *vll^-/-^* | None | Resistant to apoptosis following induction of DNA damage, upregulation of HIF target genes, slowed growth, reduced consumption of yolk | ENU (*vhl*)  ZFN (*vll*) | (Kim et al., 2020) |
